# Antisense oligonucleotide development for the selective modulation of CYP3A5 in renal disease

**DOI:** 10.1038/s41598-021-84194-w

**Published:** 2021-02-25

**Authors:** Kevin A. Lidberg, Andrew J. Annalora, Marija Jozic, Daniel J. Elson, Lu Wang, Theo K. Bammler, Susanne Ramm, Maria Beatriz Monteiro, Jonathan Himmelfarb, Craig B. Marcus, Patrick L. Iversen, Edward J. Kelly

**Affiliations:** 1 Department of Pharmaceutics, University of Washington, Seattle, WA USA; 2 Department of Environmental and Molecular Toxicology, Oregon State University, Corvallis, OR USA; 3 Department of Environmental and Occupational Health Sciences, University of Washington, Seattle, WA USA; 4 Victorian Centre for Functional Genomics, Peter MacCallum Cancer Centre, Melbourne, Australia; 5 Depto Clinica Medica, Faculdade de Medicina FMUSP, Universidade de Sao Paulo, São Paulo, Brazil; 6 Kidney Research Institute, University of Washington, Seattle, WA USA

**Keywords:** Drug discovery, Molecular biology

## Abstract

*CYP3A5* is the primary CYP3A subfamily enzyme expressed in the human kidney and its aberrant expression may contribute to a broad spectrum of renal disorders. Pharmacogenetic studies have reported inconsistent linkages between *CYP3A5* expression and hypertension, however, most investigators have considered CYP3A5*1 as active and CYP3A5*3 as an inactive allele. Observations of gender specific differences in CYP3A5*3/*3 protein expression suggest additional complexity in gene regulation that may underpin an environmentally responsive role for *CYP3A5* in renal function. Reconciliation of the molecular mechanism driving conditional restoration of functional CYP3A5*3 expression from alternatively spliced transcripts, and validation of a morpholino-based approach for selectively suppressing renal CYP3A5 expression, is the focus of this work. Morpholinos targeting a cryptic splice acceptor created by the CYP3A5*3 mutation in intron 3 rescued functional *CYP3A5* expression in vitro, and salt-sensitive cellular mechanisms regulating splicing and conditional expression of CYP3A5*3 transcripts are reported. The potential for a G-quadruplex (G4) in intron 3 to mediate restored splicing to exon 4 in CYP3A5*3 transcripts was also investigated. Finally, a proximal tubule microphysiological system (PT-MPS) was used to evaluate the safety profile of morpholinos in proximal tubule epithelial cells, highlighting their potential as a therapeutic platform for the treatment of renal disease.

## Introduction

CYP3A5 (cytochrome P450, family 3, subfamily A, polypeptide 5) is a heme-thiolate monooxygenase that oxidizes a variety of xenobiotics and steroid hormones including cortisol and corticosterone^1,2^. *CYP3A5* is the only CYP3A isoform expressed in the human kidney but genetic variants can affect expression level and activity^2–4^. Specifically, the CYP3A5*1 allele encodes a full-length, functional enzyme while the CYP3A5*3 allele contains a single nucleotide polymorphism (6896G > A) in intron 3 generating a cryptic splice acceptor and nonsense mediated decay (NMD) of the aberrant mRNA^5,6^. *CYP3A5* genotype contributes to interindividual differences in intrarenal disposition of drugs like cyclosporine and is postulated to modulate blood pressure, due to its ability to form and eliminate glucocorticoid receptor (GR) and mineralocorticoid receptor (MR) substrates influencing sodium retention^7–10^. CYP3A5 expression and activity is consistently correlated with blood pressure in animal models of hypertension^8,11–13^. However, attempts to establish a link between CYP3A5 genotype and hypertension in humans have yielded inconsistent outcomes^14–19^. The allele linkage may be complicated by the fact that CYP3A5*3/*3 individuals can sometimes express functional CYP3A5^20^. While the cellular mechanisms and environmental drivers regulating this conditional expression remain unclear, it is possible that hypertensive cohorts that include CYP3A5*1/*3 and CYP3A5*3/*3 individuals may not consistently predict outcomes associated with moderate or slow metabolizers^20,21^. The mechanism by which CYP3A5*3/*3 individuals produce functional CYP3A5 is unknown, but we hypothesized it may involve alternative splicing of precursor messenger RNA (pre-mRNA).

Alternative pre-mRNA splicing occurs in all human genes and provides functional expansion of the proteome^22,23^. For the human cytochrome P-450 (CYP) family of 57 genes, alternative splicing increases the number of unique mRNA transcripts to nearly 1000, and may represent an adaptive mechanism to chemical exposure^24^. Recently it has been discovered that polyguanine sequences are overrepresented in RNA near splice sites where they can form G-quadruplex (G4)-RNA structures capable of altering pre-mRNA splicing patterns^25^. G4 structures observed in both intronic and exonic regions can act as either splice enhancers or splice silencers^26–32^. G4 RNA formation is strongly influenced by flanking nucleic acid sequence, and the local concentrations of RNA binding proteins and both monovalent and divalent cations^33^. Potassium (K +) is maintained at levels above 100 mM in many cell types, and it is assumed to be the primary driver of G4 assembly *in vivo*^34,35^. Alternative cations including sodium (Na +) are known to stabilize different G4 structures in vitro, while lithium (Li +) can be a G4 destabilizing agent^33–36^. Because G4 RNA structures respond to transient changes in intracellular cation levels they can conditionally-modulate gene expression and splicing events, as well as the function of non-coding RNA^37^. We hypothesize that the restoration of functional CYP3A5 expression observed for CYP3A5*3/*3 polymorphs may be responsive to cellular stressors that influence renal cation storage and transport.

Here, we tested the hypothesis that a dynamic G4-motif located near the CYP3A5*3 SNP site regulates the cryptic, mutant splice acceptor in intron 3, conditionally shifting spliceosome recognition back to the wild-type exon 4 splice acceptor. These studies were performed using a CYP3A5*3/*3 confirmed HEK293 cell line, and antisense oligonucleotides (ASOs) targeting the cryptic splice acceptor site (*6986A* > *G*; *rs776746*) or a putative G4 motif in intron 3. We previously showed the utility of using ASO interference to alter spliceosome function by targeting an exon splice-acceptor site in the *c-myc* transcript^38^. This strategy has now been used to restore the reading frame in mutant forms of *dystrophin*; create a ligand independent *CTLA-4* splice variant; and modulate immune responses to Ebolavirus^39–44^. The current studies utilized synthetic oligomers to confirm the role of the *3 SNP and a putative, intron 3 G-quadruplex in enhancing the exon 4 splice-acceptor in CYP3A5*3/*3, which leads to restoration of functional enzyme expression under high salt conditions. Because some MR ligands generated by CYP3A5 promote sodium retention, renal CYP3A5 inhibition strategies may be appropriate for treating hypertension. However, most small molecule inhibitors of CYP3A5 also tend to inhibit CYP3A4, creating the potential for toxic, drug-drug interactions^45,46^. Recenlty, clobetasol propionate (clobetasol), a potent topical corticosteroid used to treat psoriasis, was identified as a potent and selective CYP3A5 inhibitor^47,48^. Here we demonstrate that CYP3A5 expression can alternatively be modulated using ASOs that interact with target RNA via Watson–Crick base pairing^49,50^.

The two chemistries usually employed for ASOs are phosphorodiamidate morpholino oligomers (PMOs) and phosphorothioate oligomers (PSOs). PSOs have phosphorothioate nucleic acid linkages with fully or partially (in the case of gapmers) substituted ribose rings at the 2′ position of various chemistry (e.g. O-methyl, OMe; O-methoxyethyl, MOE), while PMOs have phosphorodiamidate linkages and morpholine rings. These chemical modifications affect several properties including protein binding, affinity for RNA, metabolic and renal clearance, and are required to achieve a pharmacologically relevant resident time in target tissue^51–53^. PMOs and PSOs of various 2′-ribose chemistries are primarily eliminated by renal excretion, but they also accumulate in PTECs (proximal tubule epithelial cells), which can cause tubular degeneration and regeneration once the ASO surpasses a molecule-specific concentration threshold^54,55^. This is observed at lower doses for PSOs than PMOs and can be accompanied by increases in biomarkers of acute kidney injury (AKI) such as serum creatinine and urinary kidney injury molecule 1 (KIM-1). In addition, proteinuria is observed more often with PSOs than PMOs, though this may represent competitive inhibition of an uptake process^56–61^. Therefore, targeting CYP3A5 with ASOs is not without risk as nephrotoxicity is a safety liability for this class of molecules, although it may be chemistry-dependent and is usually mild and reversible^62–64^.

Screening strategies to select candidate ASOs with favorable safety profiles include in silico optimization of base sequence to maximize specificity and reduce off-target hybridization events as well as conventional toxicology studies in animals. However, ASO-induced toxicity can be sequence-dependent, making safety characterization of candidate molecules difficult^65,66^. Several candidate ASOs found to be well-tolerated in preclinical species caused AKI in first-in-human trials^67–70^. The mechanism by which ASOs cause renal injury remains largely unknown. Consequently, human-relevant in vitro cell culture models that can discriminate between safe and toxic ASOs as well as better describe and identify the molecular mechanisms of ASO-induced toxicity are needed.

We have developed a fluidic microphysiological system (MPS) populated with primary human PTECs that demonstrates many of the biochemical, synthetic, and transport activities of the proximal tubule^71,72^. PTECs within this system have promise in safety screening applications because they manifest physiological injury responses that discriminate between structurally similar compounds of differing toxicity^73,74^. Culture of PTECs in this MPS (proximal tubule-MPS, PT-MPS) enables biologically relevant cell–cell and cell–matrix interactions as well as application of shear stress, all of which are microenvironment factors that improve native cell phenotypes over conventional 2D cultures^73,75–78^. Here, we leverage this technology to compare the safety profile of a PMO to a 2′-OMe-PSO by quantifying cell associated and secreted biomarkers as well as performing global transcriptomic analyses with RNAseq.

## Results

**Figure 1:**
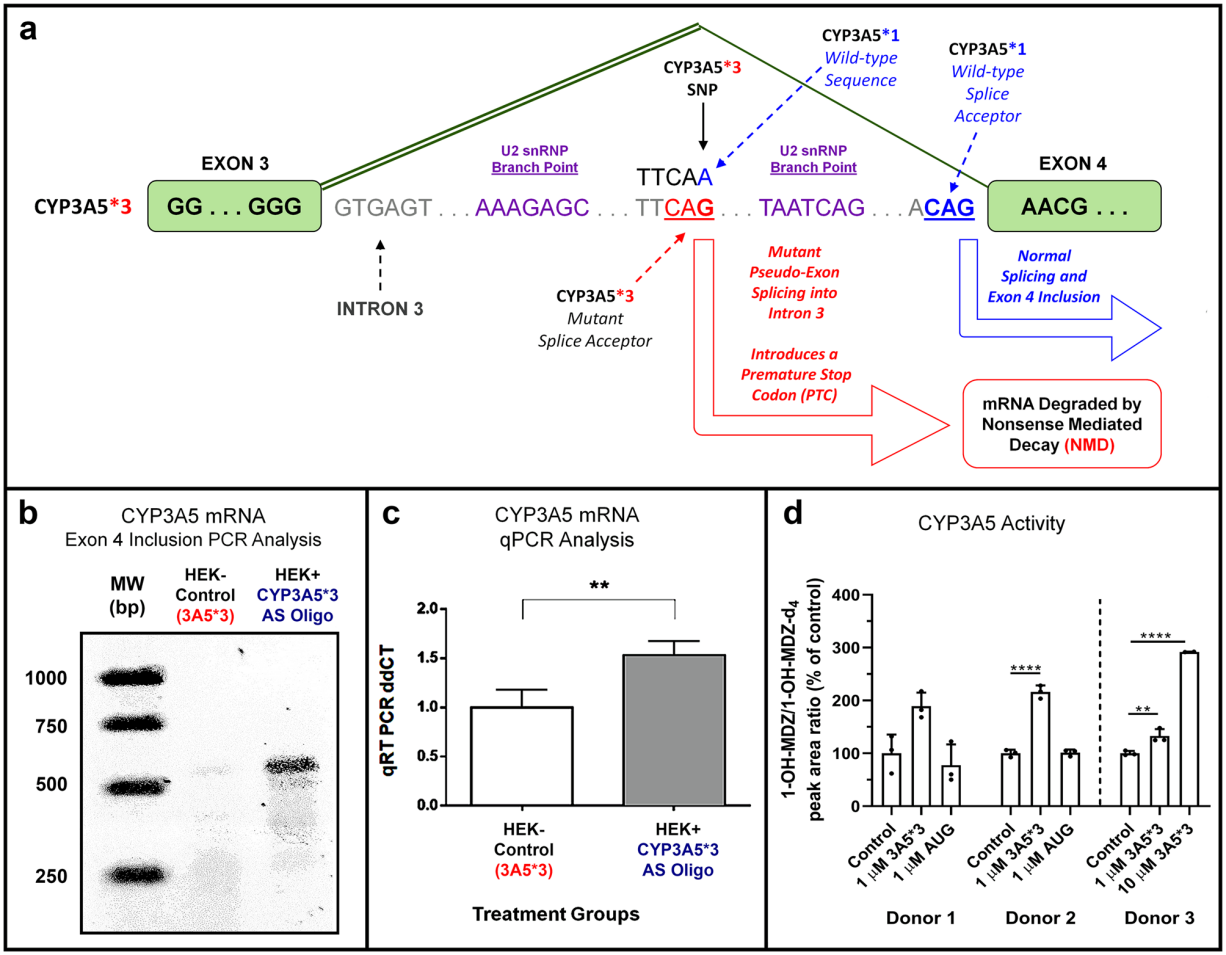
The CYP3A5*3 SNP Regulates Gene Splicing via a Salt-sensitive Mechanism that can be Modulated with ASO in Renal Cell Culture Models. (**a**) Schematic for the CYP3A5*3/*3 polymorphism found in HEK293 cells. The CYP3A5*3-SNP creates a cryptic splice acceptor within intron 3 that results in the aberrant splicing of a pseudo-exon containing a PTC that targets the mRNA transcript for degradation by NMD. Both the cryptic splice acceptor (CAG/TA) and the exon 4 splice acceptor (CAG/AA) are preceded by canonical U2 snRNP branch point *cis*-acting elements. (**b**) Endpoint PCR results monitoring exon 4 inclusion in mature *CYP3A5* mRNA transcripts demonstrate the role of the *3-SNP in altering splicing and transcript stability. Wild-type CYP3A5 mRNA transcripts encoding exon 4 are virtually undetectable (no band) in HEK293 cell lysates under normal conditions, however when the *3-SNP site is masked by interactions with an antisense oligomer (CYP3A5*3 PMO; 3 µM; 48 h) transcript stability is restored, leading to enhanced detection of exon 4 inclusion via endpoint PCR. (**c**) Quantitative PCR (qRT-PCR; SYBR green) results demonstrating a 1.5-fold increase in total *CYP3A5* mRNA when HEK293 cells were treated with the CYP3A5*3 PMO (1 µM; 48 h). (**d**) Quantification of the formation of 1-OH-midazolam from midazolam by LC/MS/MS after treatment with either 3A5*3 PMO or AUG PMO for 6 days in primary human PTECs. CYP3A5 activity was increased in PTECs treated with 3A5*3 PMO but not AUG PMO (**p < 0.01; t-test). Efficiency of restoration of CYP3A5 activity by the 3A5*3 PMO was donor-dependent (Donor 3; See Supplemental Table 1), and was not related to cellular toxicity, as PMO treatments did not alter normal PTEC morphology (see Supplemental Fig. 9).

The CYP3A5*3 variant (*rs776746*) is a common single nucleotide polymorphism (SNP) found within intron 3 of the human gene (Fig. 1a). The adenine nucleobase (*6986A/G*) altered by this mutation is highly conserved in non-human primates (Supplemental Fig. 1a), and the resulting polymorphism (*3-SNP) creates a pseudo-exon with a splice acceptor site in intron 3, with an intron/exon sequence (CAG/TA), that can compete with the reference sequence (CAG/AA) at the authentic exon 4 splice acceptor site (Fig. 1a). In addition, a U2 snRNP branch point sequence (AAAGAG) found upstream from the *3-SNP, resembles the reference branch point (AATCAG) near the exon 4 splice acceptor. Incorrect splicing of exon 4 at the *3 SNP site creates a pseudo-exon with a termination codon, located upstream of the exon 4 splice acceptor site, capable of triggering NMD and loss of the *CYP3A5* mRNA. To validate the role of the *6896A/G* polymorphism in creating the cryptic splice acceptor site in intron 3, an antisense oligonucleotide designed to bind to the SNP site was prepared, 3A5*3 PMO (Table 1). To clarify the role of the *3-SNP in promoting NMD of mature transcripts, we explored the nature of *CYP3A5* mRNA expression in a human embryonic kidney (HEK293) cell line, which was verified as homozygous *3/*3 by DNA sequencing (Supplemental Fig. 1b). Incubation of HEK293 cells with the 3A5*3 PMO (1 µM; 48 h), which masks the cryptic splice acceptor, led to increased levels of *CYP3A5* mRNA as revealed by both endpoint PCR (Fig. 1b) and quantitative (qRT) PCR (Fig. 1c). Functional restoration of CYP3A5 protein activity with the 3A5*3 PMO but not the (AUG) start site inhibitor, a PMO designed to block translation of all genotypes (Table 1), was confirmed in CYP3A5*3/*3 primary human PTECs using a midazolam metabolism assay (Fig. 1d).

**Table 1:** Antisense oligonucleotide sequences.

Name	Sequence	Target	Purpose
AUG	TTTCCCATGAGGTCCATCGCCAC	Translation start	Inhibit CYP3A5 synthesis
3A5*3	CAGGGAAGAGATATTGAAAGAC	6986A/G SNP	Shift splicing to exon 3
G4 Disrupt	CCGATTCTGCAGCTGGAGCCACAC	G4 structure in intron 3	Prevent G4 structure
E4SA-3	GAGTTGACCTTCATACGTTTCTG	Exon 4 splice acceptor	Skip exon 4; E4 EJC site
E4SA-9	TTGACCTTCATACGTTCTGTGTGGG	Exon 4 splice acceptor	Skip exon 4; E4 EJC site
E4SA-17	ACGTTCTGTGTGGGGACAACGG	Exon 4 splice acceptor	Skip exon 4; E4 EJC site
E4SA + 94	CAAAAAATGGATGCTTACCCTTCGA	Exon 4 splice acceptor	Skip exon 4
Scr	ACTCCATCGTTCAGCTCTGA	Scramble control	Negative control
HPV PMO	CCTTTAGGGTAACAAGTCTTC	Human papillomavirus	Morpholino
HPV PSO	CCTTTAGGGTAACAAGTCTTC	Human papillomavirus	2′-OMe-PSO
DSP PSO	UCAAGGAAGAUGGCAUUUCU	Dystrophin Exon 51	2′-OMe-PSO

**Figure 2:**
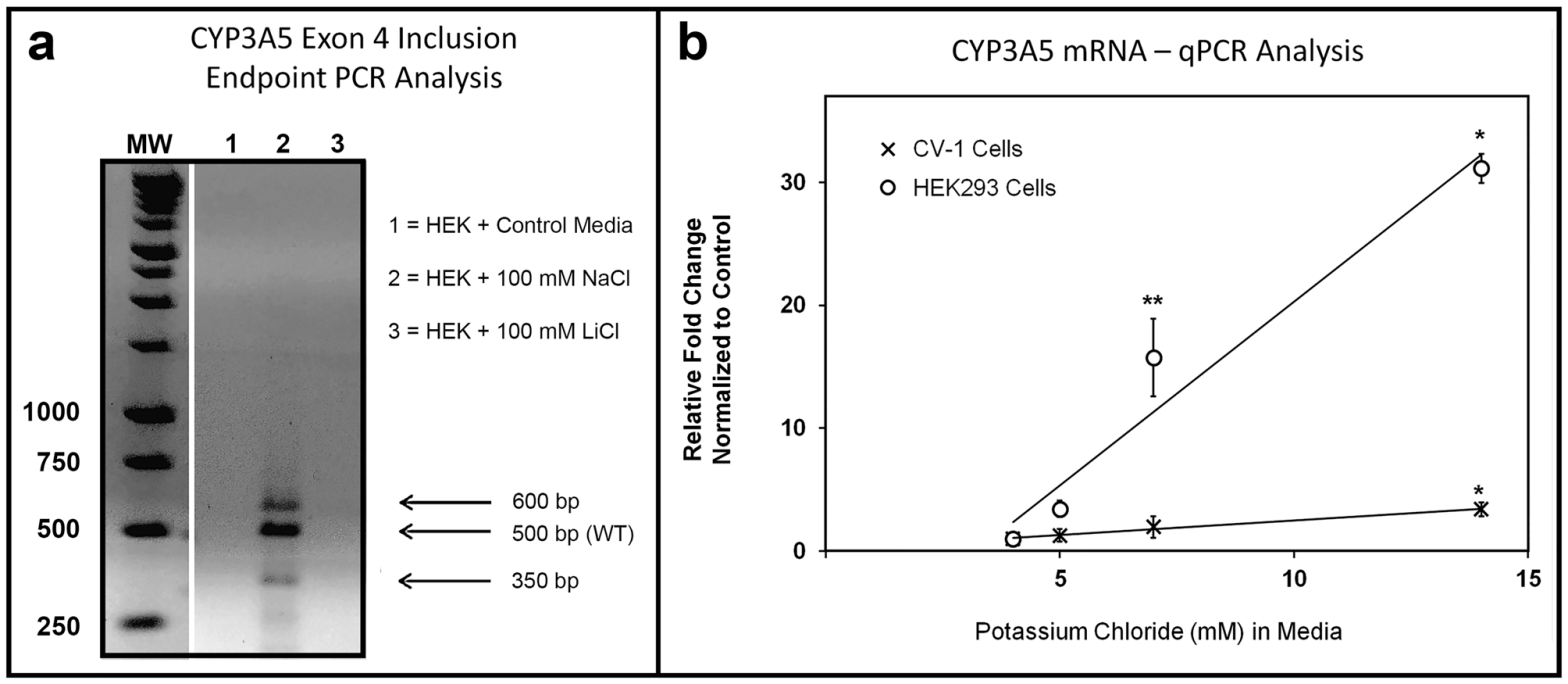
Cation-specific Restoration of CYP3A5*3/*3 mRNA in HEK293 cells, but not CYP3A5*1/*1 CV-1 cells. (**a**) Endpoint PCR monitoring of *CYP3A5* mRNA expression in HEK293 cells exposed to: 1 (control), 2 (NaCl enriched), and 3 (LiCl enriched) media revealed cation-specific induction of alternative gene splicing. Several lanes were removed from this original gel image to aid in the molecular weight interpretation shown here, and this gap is denoted by the white dividing line. (**b**) SYBR Green qPCR Analysis of *CYP3A5* mRNA from HEK293 (CYP3A5*3) and CV-1 (CYP3A5*1) cells incubated with media supplemented with increasing amounts of (4, 5, 7, and 14 mM) of KCl. Normal DMEM media contains 4 mM KCl.

While the mechanism by which CYP3A5*3/*3 individuals produce functional CYP3A5 remains unknown, our results with the 3A5*3 PMO showed that blocking the *3-*SNP* site can correct splicing and restore CYP3A5 activity in HEK293 cells. We hypothesized that a salt-sensitive, G4 structure located in proximity to the *3-SNP site might play a similar *“trans-acting*” role in driving alternative splicing of the pre-mRNA transcript. Computational analysis of the human CYP3A5 intron 3 gene structure identified multiple G4 targets in a repeating triplet G sequence spanning 151 bases located between the *3-SNP and the exon 4 splice acceptor. A QGRS computational search of this region identified the signature of two conserved G4 structures at the 3′ end of intron 3 (see Supplemental Fig. 2)^79^. To validate the hypothesis that a G4 structure participates in *CYP3A5* splicing and NMD suppression in CYP3A5*3 genotypes, we tested the effect of G4-stabilizing, Na + cations on pre-mRNA splicing in cultured HEK293 cells. RNA extracted from HEK293 cells grown in standard media failed to produce a visible band indicative of normal exon 4 inclusion (Fig. 2a), while a visible band of expected size (500 bp) was present in RNA extracted from cells grown in hyperosmolar media (supplemented with 100 mM NaCl). Two minor bands at 350 and 600 bp were also present in the PCR reaction but were not considered diagnostic for exon 4 inclusion. It is possible that these bands indicate additional complexity in exon 4 splicing under high salt conditions, but further analysis is required to rule out off-target PCR artifacts (Fig. 2a). A tonicity-responsive enhancer element located in intron 2 of the *CYP3A7* gene, which regulates CYP3A family gene expression, has been previously described^80^. To discriminate the role of this *cis* acting element from the putative G4 element in intron 3 of *CYP3A5*, we supplemented HEK293 media with G4-destabilizing, Li + cations (LiCl; 100 mM) to raise osmolarity while destabilizing any G4 structures in the pre-mRNA transcript. Exon 4 inclusion bands were not observed in HEK293 cells treated with LiCl, indicating that splice correction in *3/*3 mRNA transcripts is cation-specific, and not simply a function of tonicity, further supporting a G4 element in proximity to *3-SNP of *CYP3A5* (Fig. 2a). To further establish the discrete role of the *3-SNP in mediating, cation-specific modulation of *CYP3A5* expression in HEK293 cells, we utilized qPCR to compare the effects of hyperosmolarity on a related mammalian cell type (CV-1 cells; African Green Monkey Kidney Fibroblasts) expressing the CYP3A5*1 genotype (Fig. 2b); genomic sequencing has confirmed the green monkey does not express the human (*6986A/G*) SNP, similar to other non-human primates (Supplemental Fig. 1c). The relative fold change in wild-type *CYP3A5* mRNA expression was monitored in both HEK293 and CV-1 cells using the 2^−ΔΔCT^ method^81^. KCl supplementation (of 1, 2 and 10 mM to DMEM media containing 3 mM KCl) had minimal influence on CYP3A5 expression in CV-1 cells, but significantly induced CYP3A5 mRNA transcript levels in HEK293 cells (** *p* < 0.01; * *p* < 0.05; t-test), in a dose-dependent manner that supports the participation of a G4 structure (Fig. 2b).

**Figure 3:**
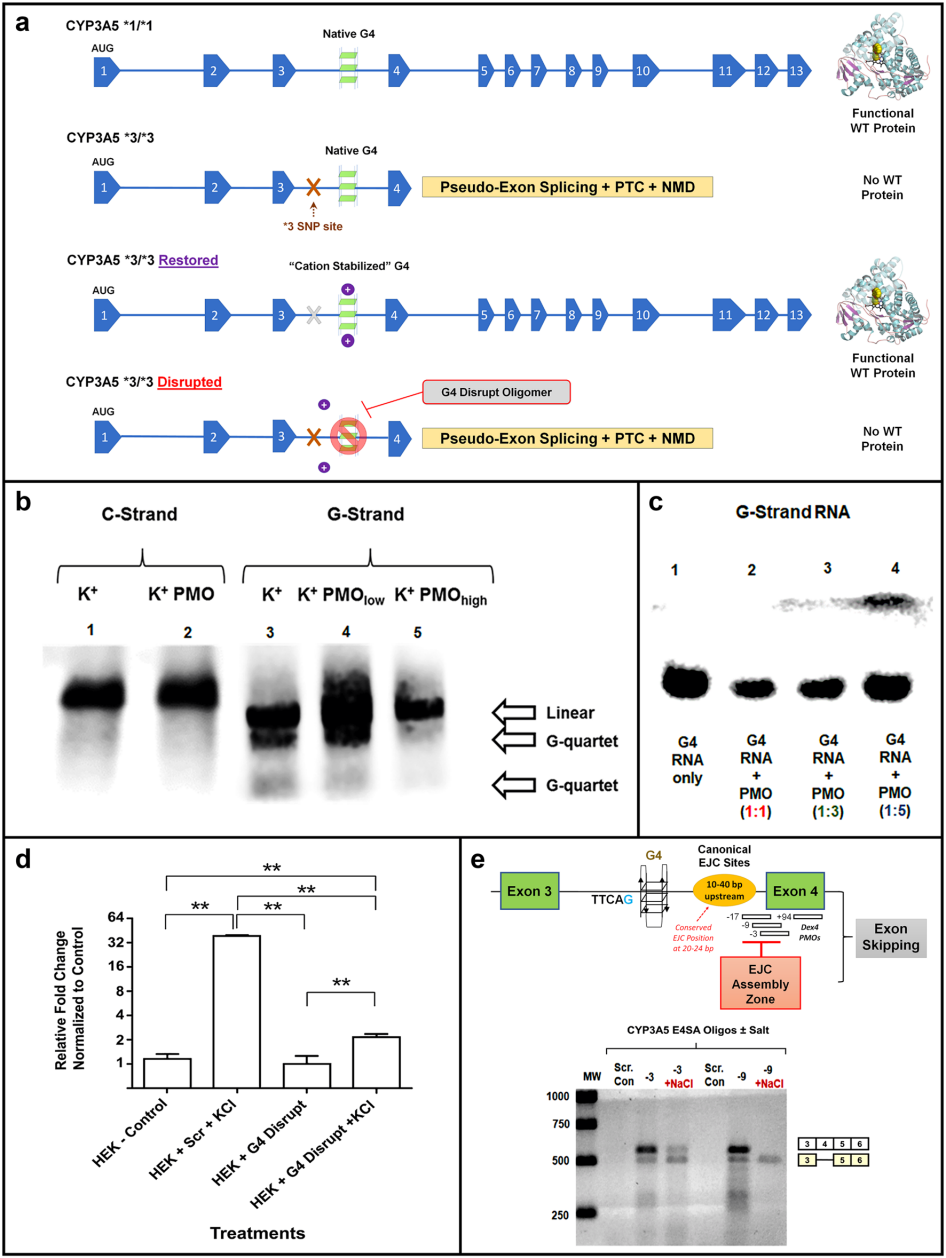
G4 Structures Regulate CYP3A5 Expression and Represent Novel Targets for ASO Modulation. (**a**) Schematic diagram of the CYP3A5 gene showing the location of a putative G4 motif in Intron 3. Cation-specific stabilization of native G4 structure shifts the splice acceptor back to the reference exon 4 splice acceptor, creating an in frame CYP3A5*3/*3 transcript that bypasses pseudo-exon introduction and suppression of wild-type protein translation via PTC or NMD-related mechanisms. Salt-sensitive splice correction of *3 transcripts (shown in Fig. 2a) can be abrogated using the G4 disrupting PMO (Table 1). (**b**) Computational analysis of the CYP3A5 gene (see Supplemental Fig. 2) and RNA/DNA gel shift assays (*shown here*), confirmed the existence of a G4-like structure in the CYP3A5 intron 3 region. Control lanes 1 and 2 depict the mobility of the 151 nucleobase, CYP3A5 intron 3 “C-strand” DNA, in KCl-enriched running buffer [100 mM KCl]. The C-strand does not display faster migration typical of a G4 structure in the presence of KCL, and a G4 disrupting PMO does not alter C-strand mobility (lane 2). In contrast, the 151 nucleobase “G-strand” DNA demonstrated enhanced mobility bands reflecting both inter- and intra-strand G4 formation in KCl-enriched running buffer (lane 3). Addition of the G4 disrupting PMO diminishes the enhanced mobility bands at low concentration (1:1 molar ratio; lane 4) and ablates the enhanced mobility bands at high concentration (5:1 molar ratio; lane 5). (**c**) A 151 nucleobase RNA strand also forms a putative G4-structure in KCl-enriched running buffer (Lane 1). Addition of increasing amounts of G4 disrupting PMO to the G4 RNA (Lane 2 – 1:1 PMO:RNA ratio, Lane 3 – 3:1 PMO:RNA ratio, and Lane 4 – 5:1 PMO:RNA ratio) shifts the RNA structure to the slower mobility form. (**d**) qPCR analysis of CYP3A5 mRNA from HEK293 cells exposed to no PMO and normal media (HEK control), scramble PMO (1 µM, 48 h.) and KCl (12 mM) (HEK + Scr + KCl), G4 disrupting PMO only (1 µM, 48 h.) (HEK + G4 Disrupt), and G4 disrupting PMO (1 µM, 48 h.) and KCl (12 mM) (HEK + G4 Disrupt + KCl). Significant statistical differences were monitored by ANOVA; *p* value < 0.01. Post test *p* < 0.01 = **. (**e**) (*Top*) A schematic illustrating the location of the EJC in relation to the cryptic splice acceptor in intron 3 and the G4 structure. PMOs designed to skip exon 4 in CYP3A5 are also shown (-17 = E4SA-17, -9 = E4SA-9, -3 = E4SA-3, and + 94 = E4SA + 94). (*Bottom*) Endpoint PCR results for RNA expression in HEK293 cells treated with three PMOs (E4SA-3, E4SA-9, or a scramble control (Scr); 1 µM; 48 h.) and 100 mM NaCl are shown. The Scr. oligomer did not induce stable transcripts, while exon 4 skipping PMOs (E4SA -3 and -9) induced transcripts that both include and exclude exon 4. The addition of high salt media diminished the cell’s ability to express transcripts including exon 4. Cationic-specific stabilization of the transcript may have improved the ability of exon skipping PMOs to properly bind the transcript near the EJC assembly zone and promote targeted exon 4 exclusion. Molecular drawings were generated using The PyMOL Molecular Graphics System, Version 1.3, Schrödinger, LLC.

A model depicting G4 structures in *CYP3A5* intron 3 that modulate exon 4 splicing event*s* is shown in Fig. 3a. Cation-specific G4 stabilization of *3-SNP transcripts may shift the splice acceptor back to the reference exon 4 splice acceptor creating viable mRNA that bypasses pseudo-exon splicing and PTC incorporation that leads to altered translation of wild-type CYP3A5 or NMD of mRNA transcripts. We predicted ASOs targeting the G4 motif (called G4 Disrupt; Table 1) could prevent NMD escape and limit the cation-specific restoration of *3/*3 transcripts in HEK293 cells. To test whether the proposed G-rich motifs in intron 3 could fold into G4-like structures in vitro, oligomeric DNA and RNA spanning the 151 base G-rich region of intron 3 was prepared (Table 2). Equal length oligomers containing the complementary C-rich sequences were used as controls. The DNA oligomers were incubated in the presence of 100 mM KCl and resolved on a native PAGE gel (Fig. 3b, Lanes 1–5). Multiple, differentially migrating bands were observed in lanes with the “G strand” DNA oligomer from intron 3 (Fig. 3b, Lanes 3–5); these bands were absent in matched “C-strand” experiments (Fig. 3b, Lanes 1–2). The G4 Disrupt PMO, with sequence complementary to the G-rich motifs identified by QGRS, had no effect on the C-strand mobility, but at high concentrations (fivefold molar excess) ablated the faster migrating bands in the G-strand (Fig. 3b, Lane 5). An RNA oligomer with sequence equivalent to the intronic DNA oligomer was used to establish G4 characteristics in the related RNA structure. Nearly all the intronic RNA formed faster migrating intra-strand G4 structures in the presence of KCl (Fig. 3c, Lane 1). Incubation with 1:1, 3:1, and 5:1 (G4 Disrupt) PMO to RNA ratios led to an increasing shift from fast migrating G4-like structures to the slower migrating linear form (Fig. 3c, Lanes 2–4).

**Table 2:** DNA and RNA oligomers used in gel shift mobility assays.

Name	Sequence
*HOX11* G-strand—DNA oligomer sequence:	5′-GCG CGA GGG AGG GGA GGG GAG GGG GAG AGG-3′
*HOX11* C-strand—DNA oligomer sequence:	5′-CCT CTC CCC CTC CCC TCC CCT CCC TCG CGC-3′
*CYP3A5_intron3_*G-strand—DNA oligomer sequence:	5′-GGG TGG CTC CTG TGT GAG ACT CTT GCT GTG TGT CAC ACC CTA ATG AAC TAG AAC CTA AGG TTG CTG TGT GTC GTA CAA CTA GGG TCG TAT GGA TTA CAT AAC ATA ATG ATC AAA GTC TGG CTT CCT GGG TGT GGC TCC AGC TGC AGA ATC GGG-3′
*CYP3A5_intron3_*C-strand—DNA oligomer sequence:	5′- CCC GAT TCT GCA G CT GGA GCC ACA CCC AGG AAG CCA GAC TTT GAT CAT TAT GTT ATG TAA TCC ATA CCC CTA GTT GTA CGA CAC ACA GCA ACC TTA GGT TCT AGT TCA TTA GGG TGT GAC ACA CAG CAA GAG TCT CAC ACA GGA GCC ACC C-3’
*CYP3A5_intron3_*G-strand—RNA oligomer sequence:	5′-GGG UGG CUC CUG UGU GAG ACU CUU GCU GUG UGU CAC ACC CUA AUG AAC UAG AAC CUA AGG UUG CUG UGU GUC GUA CAA CUA GGG UCG UAU GGA UUA CAU AAC AUA AUG AUC AAA GUC UGG CUU CCU GGG UGU GGC UCC AGC UGC AGA AUC GGG-3′

Next, we tested the ability of the G4 Disrupt PMO to suppress cationic rescue of *3/*3 transcripts in HEK293 cells incubated with KCl (Fig. 3d). Incubation with the G4 Disrupt PMO significantly blocked salt induction of viable CYP3A5 transcripts in HEK293 cells (48 h., 1 µM; as compared to the scrambled (Scr) PMO control (Table 1); *p* < 0.01) (Fig. 3d). Interestingly, CYP3A5 exon 4 skipping PMOs (Table 1) designed to target the *CYP3A5* intron 3/exon 4 border may also alter the assembly of the exon junctional complex (EJC), as canonical eIF4AIII binding sites have been identified near their target site, ~ 10–40 bp upstream of the splice junction, with a conserved EJC target site at 20–24 bp upstream^82,83^. Dex4 oligomers that bind downstream of the EJC, but not exclusively within exon 4 (Exon 4 Splice Acceptor PMOs (E4SA) -3 and -9, but not − 17 and + 94; see Table 1), were able to induce both wild-type and delta exon 4 (Dex4) *CYP3A5* mRNA expression in HEK293 cells, without NaCl supplementation to the cell culture media (Fig. 3e). High salt levels appear to have altered the interaction of E4SA PMOs with target sequences near the EJC assembly zone, reducing the cationic-specific suppression of pseudo-exon inclusion and NMD, while enhancing the targeted skipping of exon 4 (Fig. 3e). These results highlight the dynamic splice sensitivity of CYP3A5*3 transcripts, that we attribute in part to a newly identified G4 element in intron 3. While more advanced biophysical methods (e.g., circular dichroism or NMR) are needed to fully characterize this theoretical secondary structural element, our data strongly support the existence of a conserved G4 element in intron 3 that alters the phenotypic presentation of CYP3A5*3 genotypes via salt-sensitive, alternative splicing and NMD suppression.

**Figure 4:**
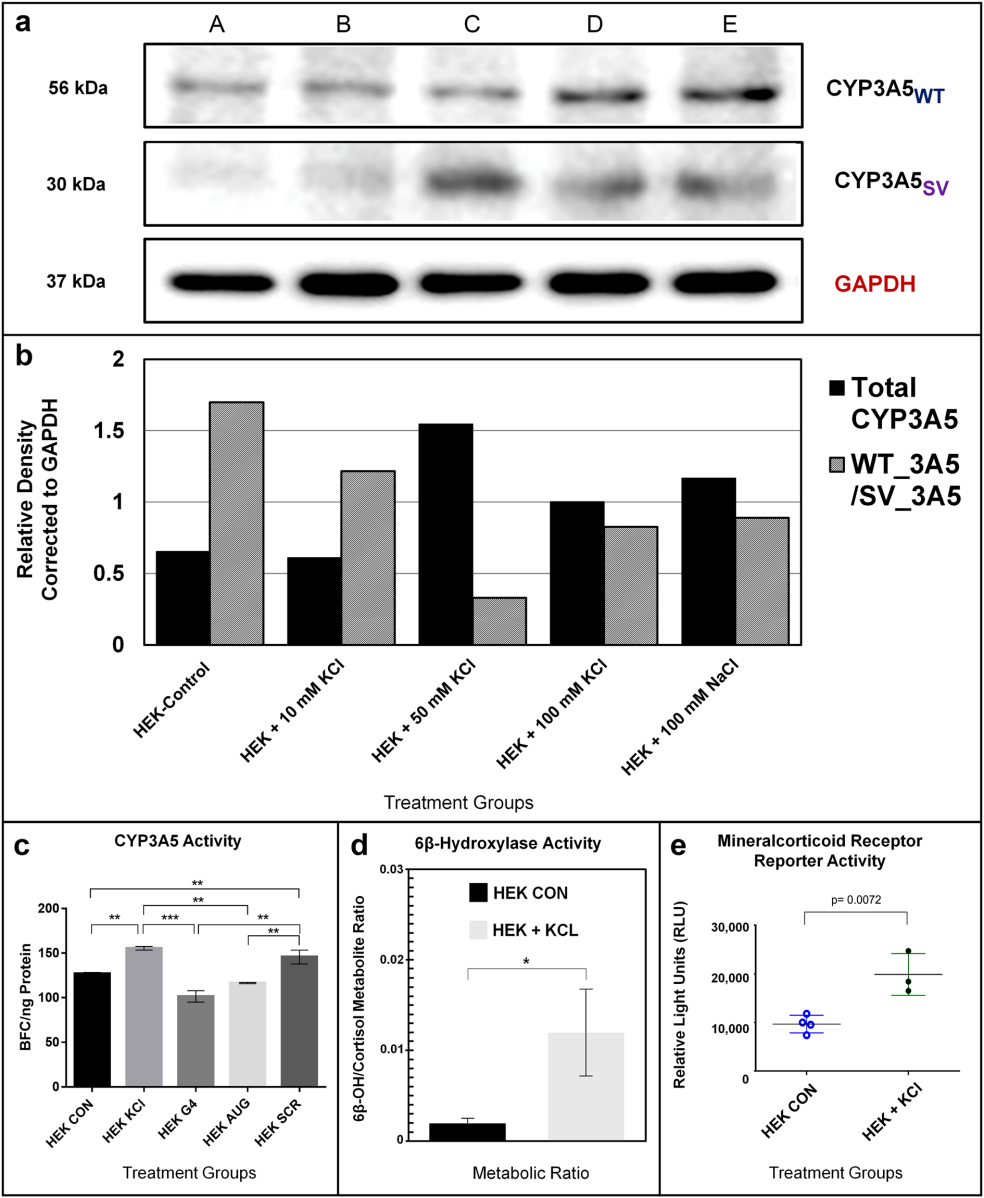
Salt-Induced CYP3A5*3 Protein Expression, Enzyme Activity and Mineralocorticoid Receptor Signaling in HEK293 Cells. (**a**) Confluent HEK293 cells were exposed for 48 h to (A) control media or media supplemented with (B) 10 mM KCl, (C) 50 mM KCl, (D) 100 mM KCl, or (E) 100 mM NaCl. Two protein bands attributed to CYP3A5 were detected via western blot, including the full-length reference protein (*CYP3A5*_*WT*_) at 56 kilodaltons (kDa) and a truncated 30 kDa splice variant form (*CYP3A5*_*sv*_). The housekeeping gene *GAPDH*, detected at 37 kDa, was used to normalize sample loading. (**b**) Total *CYP3A5* protein and the ratio of CYP3A5_WT_ to CYP3A5_SV_ protein normalized to GAPDH expression was determined using densiometric analysis in ImageJ. (**c**) CYP3A enzyme activity was monitored in HEK293 cells under normal conditions and with elevated KCl (12 mM; 48 h.) and PMOs (1 µM; 48 h.), as measured fluorometrically by BFC metabolism (see “Methods”). KCl supplementation significantly induced enzyme activity (*p* < 0.01; t-test) in HEK293 cells (KCl) compared to control cells (CON). The G4 disrupting PMO (G4) and the *CYP3A5* start-site inhibitor (AUG) PMO both significantly prevented KCl induction of BFC metabolism (*p* < 0.01 compared to KCl treated control) and were not different from control cells. The scrambled (SCR) PMO control did not inhibit KCl induction of CYP3A activity (ANOVA *p* value < 0.01. Post test *p* < 0.01 = **, and *p* < 0.005 = ***). (**d**) LC MS/MS analysis of HEK293 cell media demonstrated KCl enrichment (12 mM, 24 h) rapidly stimulated the conversion of cortisol to 6β-hydroxycortisol. An ~ sixfold change in the metabolic ratio was observed for HEK293 cells grown in normal media (1.93 × 10^−3^, solid black) compared to 12 mM KCL-supplemented media (1.19 × 10^−2^, solid gray). (**e**) Conditioned media from HEK293 cells (+ /- KCl (12 mM); 48 h.) were added to a luciferase-based cell reporter system for mineralocorticoid receptor (MR) transactivation (IB00501-32; Indigo Biosciences). MR activity was significantly elevated > two-fold (*p* < 0.01; t-test) in HEK293 media treated with 12 mM KCl. The ligand-specific sensitivity of the MR activity assay was validated using a standard curve for aldosterone (1 nM sensitivity =  ~ 100,000 RLU).

Western blot analysis was next used to confirm that in-frame CYP3A5*3 transcripts, rescued from NMD by NaCl or KCl, can be translated into viable protein in HEK293 cells (Fig. 4a). We observed two CYP3A5 protein bands in our western blots, corresponding to the ~ 56 KDa reference protein, and a shortened, ~ 30 KDa splice variant. Total CYP3A5 protein expression increased when HEK293 cells were treated with increasing KCl (10–100 mM) or 100 mM NaCl (Fig. 4a,b, Lanes A–E), however the ratio of wild type to variant protein was reduced in all salt treatments compared to the HEK control (Fig. 4b, Lanes B–E). The salt-induced expression of both reference and variant *CYP3A5* protein in HEK293 cells further supports the hypothesis that a G4 structure can masks the cryptic splice acceptor formed by the *3-SNP and alter the structure of the EJC at the CYP3A5 intron3/exon 4 border. Two models to explain this alternative splicing, where the G4 motif serves as either an internal ribosomal entry site (IRES) or an exonic splicing enhancer (ESE) element that can influence the availability of translational initiation start sites, are proposed in Supplemental Fig. 3.

The functional activity of salt-induced *CYP3A5* expression was next confirmed by observing CYP3A-specific metabolic activity in HEK293 cells based on the conversion of 7-benzyloxy-4-[trifluoromethyl]-coumarin (BFC) to 7-hydroxy-4-[trifluoromethyl]-coumarin (HFC). As shown in Fig. 4c, HEK293 cells treated with KCl (12 mM; 48 h.) showed a significant increase in CYP3A enzyme activity that was not suppressed when cells were co-treated with a scramble control (Scr) PMO (*p* < 0.01; t-test). In contrast, co-treatments with either the G4 Disrupt PMO or the start-site targeting (AUG) PMO (Table 1) significantly reduced CYP3A enzyme activity to levels observed for untreated HEK293 cells. *CYP3A5* is also known to catalyze the endogenous conversion of glucocorticoids, including cortisol and corticosterone, to 6β-hydroxylated products that stimulate mineralocorticoid receptor (MR) activity. LC/MS/MS analysis was used to confirm the HEK293 cell-based conversion of parent cortisol to 6β-hydroxycortisol (6βOH) metabolites (see “Methods”). As shown in Fig. 4d, HEK293 cells incubated in normal media supplemented with cortisol (1 nM; 24 h) produced sixfold less 6β-hydroxycortisol metabolite (AUC = 1.93 × 10^−3^ ± 0.05 × 10^−3^) compared to HEK293 cells cultured in KCl-enriched media (12 mM; AUC = 1.19 × 10^−2^ ± 0.48 × 10^−2^; * < 0.05; *p* = 0.031; t-test). Enhanced 6β-hydroxycorticosteroid production was subsequently confirmed to significantly increase MR signaling activity (more than twofold) in a luciferase-based cell reporter system (*p* < 0.01; t-test; see “Methods”) (Fig. 4e).

**Figure 5:**
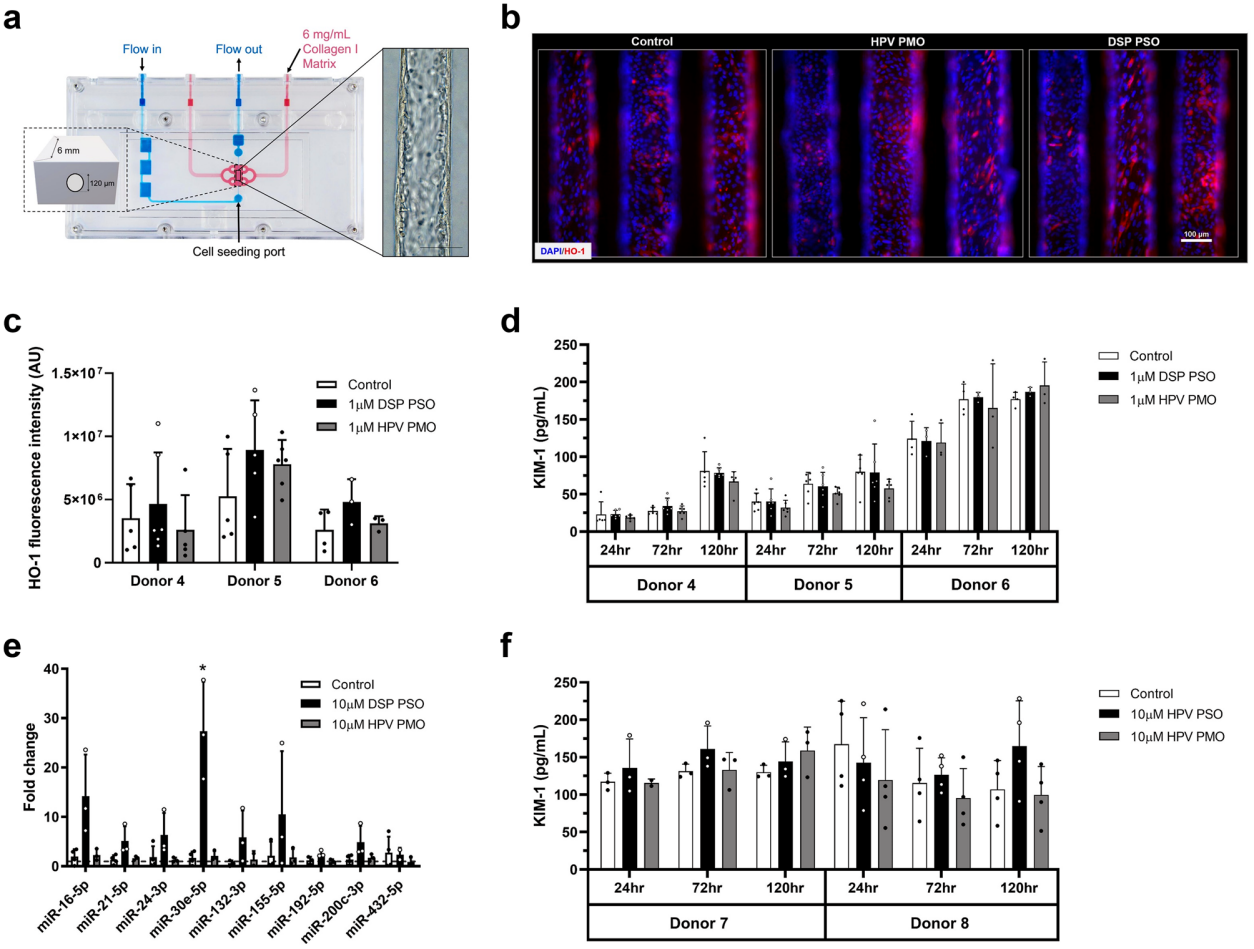
Safety Evaluation of ASO chemistry in PT-MPS. (**a**) Schematic of the PT-MPS with a phase contrast image of a confluent PTEC tubule. (**b**) Representative images of tubules stained for HO-1 (red) by immunocytochemistry after 5 days of treatment with 1 µM HPV PMO or 1 µM DSP PSO (scale = 100 µm). Cells display a predominately perinuclear and cytoplasmic HO-1 signal. (**c**) Quantification of HO-1 fluorescence intensity from tubules depicted in B. Expression and localization of HO-1 is not significantly affected by HPV PMO or DSP PSO treatment. (**d**) Quantification of levels of KIM-1 in device effluent over time by mesoscale chemiluminescent assay after treatment with 1 µM HPV PMO or 1 µM DSP PSO. There is a significant reduction in KIM-1 secretion with PMO treatment compared to control (*p* = 0.043, ANOVA with Tukey’s adjustment) and PSO groups (*p* = 0.01, ANOVA with Tukey’s adjustment) (**e**) Quantification of levels of a panel of renal disease- and injury-associated miRNAs by qPCR after treatment with 10 µM HPV PMO or 10 µM DSP PSO for 44 h. Data are presented as the delta-deltaC_T_ fold change in miRNA levels relative to control. Secretion of miR-30e-5p (*p* = 0.024, ANONVA, with Tukey’s adjustment) was significantly increased by DSP PSO treatment. (**f**) Quantification of levels of KIM-1 in device effluent over time by ELISA after treatment with 10 µM HPV PMO or 10 µM HPV PSO. There was no time- or dose- dependent effect of HPV PMO, DSP PSO, or HPV PSO treatment on the secretion of KIM-1. For all quantitative panels (C-F), data are presented as mean ± s.d with black circles representing the value for each individual tubule tested.

Given that *CYP3A5* activity may exacerbate high blood pressure (HBP) in both fast metabolizing *1/*1 and slow metabolizing (or conditional) *1/*3 and *3/*3 genotypes, and this pathological activity can be modulated with ASO in vitro, developing *CYP3A5*-directed therapeutics targeting renal disorders is worth consideration. To advance in vivo development of a CYP3A5-specific antisense inhibitor, we next utilized our human kidney PT-MPS to evaluate whether ASO safety towards target PTECs would depend on the charge of the antisense backbone chemistry (e.g., neutral PMO or (−) charged PSO) (Fig. 5a). Hemeoxygenase-1 (HO-1) is strongly induced in PTECs after exposure to nephrotoxic compounds, including those that promote generation of reactive oxygen species, making it a good biomarker for this safety study^74^. KIM-1 is a widely-accepted urinary biomarker of PTEC injury, as it is highly upregulated in vivo following AKI with subsequent shedding of the ectodomain into urine^84^. KIM-1 outperforms traditional markers of renal function such as serum creatinine and blood urea nitrogen in early detection of injury^85^. Alternative biomarkers include a panel of miRNAs that are associated with various renal disease pathologies or injury^86,87^. Therefore, we first assessed whether HO-1 expression in the human kidney PT-MPS was affected by treatment (5 days; 1 µM) with either a model PSO (Drisapersen, DSP) or model PMO targeting the human papillomavirus (HPV) (Table 1). Staining for HO-1 by immunocytochemistry showed an expected perinuclear and cytoplasmic localization that was unchanged by treatment (Fig. 5b). Neither the DSP PSO nor HPV PMO significantly affected the expression of HO-1 while controlling for donor effect (Fig. 5c), indicating there was no change in intracellular redox environment. KIM-1 secretion was significantly lower in the PMO group (Fig. 5d) compared to control while controlling for time and donor effect (*p* = 0.043, ANOVA, Tukey’s multiple testing correction) and PSO while controlling for time and donor effect (*p* = 0.01, ANOVA, Tukey’s multiple testing correction). However, the reduction was quite modest, suggesting both ASO chemistries were well tolerated in the PT-MPS when dosed at 1 µM for a duration of 5 days. Next, we tested whether a KIM-1 response could be observed at a higher concentration (10 µM) in base sequence matched ASOs (HPV PMO and HPV PSO). No treatment related change was observed while controlling for time and donor effect (Fig. 5f). However, secretion of miR-30e-5p is significantly increased by the DSP PSO (*p* = 0.024, ANOVA, Tukey’s multiple testing correction) but not HPV PMO when treated at 10 µM for 44 h (Fig. 5e). RNAseq of total RNA from this experiment revealed the DSP PSO significantly affected the expression of 18 genes relative to control (Table 3), while the HPV PMO did not significantly affect the expression of any genes. DSP PSO treatment mainly affected genes associated with ribosome biogenesis, transcript splicing, or inflammatory response. Several members of the small nucleolar RNA C/D box 3 (SNORD3 or U3) family, which direct post-transcriptional cleavage of ribosomal precursor RNA and are required for proper biogenesis of the small ribosomal subunit, were increased^88^. Other genes that were induced include WDR74 and RNA5S9, which function in assembly of the large ribosomal subunit while RNVU1-7, RNVU1-18, and CLK1 regulate transcript splicing.

**Table 3:**
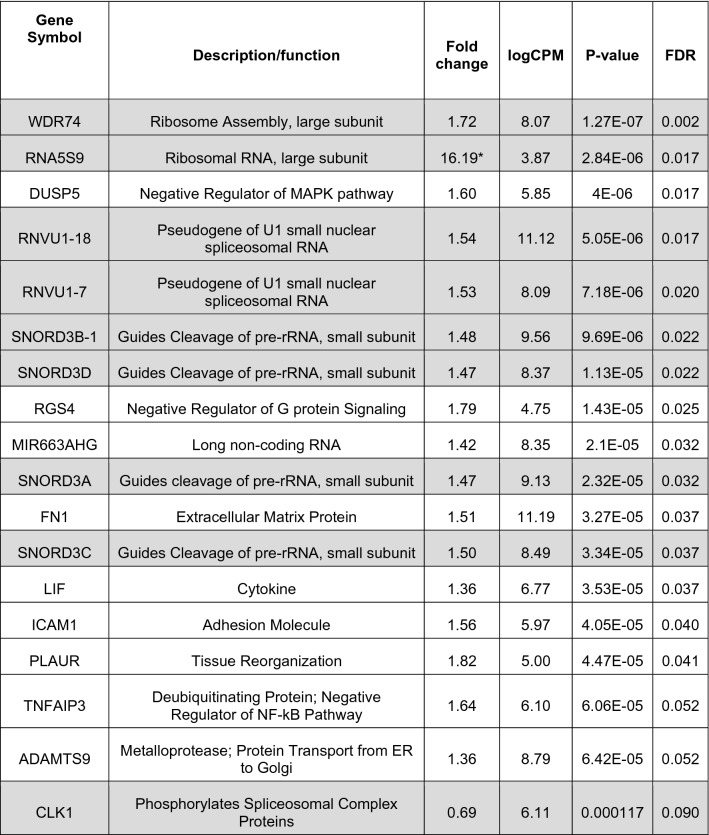
Transcriptional response of PTECs in PT-MPS to DSP PSO treatment.

Shaded rows depict genes that have functions related to nucleoprotein activity; log counts per million reads (LogCPM) is the average expression level for each gene across all samples; false discovery rate (FDR).

## Discussion

The genetic basis of disease has long been recognized, but the molecular mechanisms driving specific disorders can be challenging to interpret and characterize. Polymorphisms in both coding and non-coding RNA can cause unpredictable effects on gene expression, and their effects on gene splicing is increasingly recognized as a primary driver of multiple diseases^23,24^. A classic example of this phenomenon are SNPs in the mouse SCNM1 gene, a splicing regulator of the neuronal sodium channel gene Scn8a, whose SNP-related splicing variant can convert mild-movement disorders into paralysis by simply shifting the splicing pattern of its target gene Scn8a, which is also sensitive to SNP-related alternative splicing of its own sequence^89^. While mutations in splicing regulators like SCNM1 have the potential to cause disease by altering both targeted and global splicing events in a cell, SNPs that alter the expression of metabolic genes like cytochromes P450 can also have profound pleiotropic effect on host metabolism and gene expression networks that drive disease. In this regard, SNPs in the CYP3A5 gene have been extensively studied, due to their tendency to suppress gene expression and promote renal disorders^2–21^. However, CYP3A5*3/*3 individuals harboring a null phenotype (*6986A* > *G*) are reported to unpredictably express wild-type *CYP3A5 activity,* presumably due to conditional correct intron 3 splicing^20^. While the mechanisms driving interindividual variability in CYP3A5*3/*3 wild-type protein expression remain unknown, particularly in renal tissues, gender specific differences in sex or dietary hormones, like estrogen, vitamin A or the vitamin D hormone, have been postulated^20,90^. Building off of observations that CYP3A5 gene expression is also salt-sensitive, we wanted investigate the possibility that dietary salts could also regulate the alternative splicing of CYP3A5*3/*3 transcripts and help explain conflicting reports that CYP3A5 is involved in renal diseases like hypertension^80^. We also wanted to explore the mechanistic basis of alternative splicing in CYP3A5, using antisense oligonucleotides targeting the *3-SNP site and other secondary structural elements in close proximity that may act in cis or trans to alter the splicing and expression of CYP3A5*3/*3 transcripts*.*

Here we demonstrated that blocking the *3-SNP with a PMO oligomer can prevent the alternative splicing and NMD of CYP3A5*3 transcripts. We also investigated the possibility that a G-quadruplex (or G4) structure located near the*3-SNP site could play a salt-sensitive role in shifting spliceosome activity from the mutant splice acceptor site in intron 3 to the canonical exon 4 splice acceptor. Two conserved G-quadruplex candidates were identified in intron 3 leading to our confirmation of G4-structural character in a 151-base-region between the CYP3A5*3 SNP site and the exon 4 splice acceptor. G4 assembly was facilitated by Na + and K + cations, but not Li + cations, consistent with previously reported G4-structural behavior^33–36,91,92^. The action of G4 motifs in modulating pre-mRNA splicing is well established, therefore it is not surprising to observe a G4-mediated splicing shift in CYP3A5*3/*3 pre-mRNA transcripts to a mature mRNA, which is translated into a functional protein.

When a termination codon (UAA, UAG, or UGA) enters the ribosomal A site, translation termination occurs; when termination precedes an EJC, NMD is initiated^93^. The cryptic splice acceptor in intron 3 created by the *6986A* > *G* mutation leads to a premature termination codon (PTC) upstream of the exon 4 EJC, which explains why *CYP3A5* mRNA is diminished in *3/*3 variants (Figs. 1b,c and 2a). However, an ASO (CYP3A5*3 PMO) targeting the *3 SNP site in *CYP3A5*′s intron 3 can mask the mutant splice acceptor site and promote normal pre-mRNA processing (Fig. 1b,c). We hypothesized that spliceosome accessibility at the *3 SNP site might similarly be modulated by G4-forming motifs found within intron 3 of the human *CYP3A5* gene (Fig. 3a and Supplemental Fig. 2).

The kinetic evaluation of G-quadruplex formation indicates rapid nuclear formation of these secondary structures in the transcribed *CYP3A5* pre-mRNA (Fig. 2b,c). The G4-like structure in intron 3 appears to efficiently recruit *trans*-acting splicing factors, including hnRNPs and SR proteins, because the splicing shift from the *6986G* > *A* site to exon 4 splice acceptor is mediated by a relatively small number of bases. These studies investigated the behavior of the putative G4-structure when exposed to biologically feasible monovalent cation concentrations. However, the mechanisms regulating the transport of cations into the nucleus where splicing occurs, particularly in cultured PTECs, remains unresolved.

**Figure 6:**
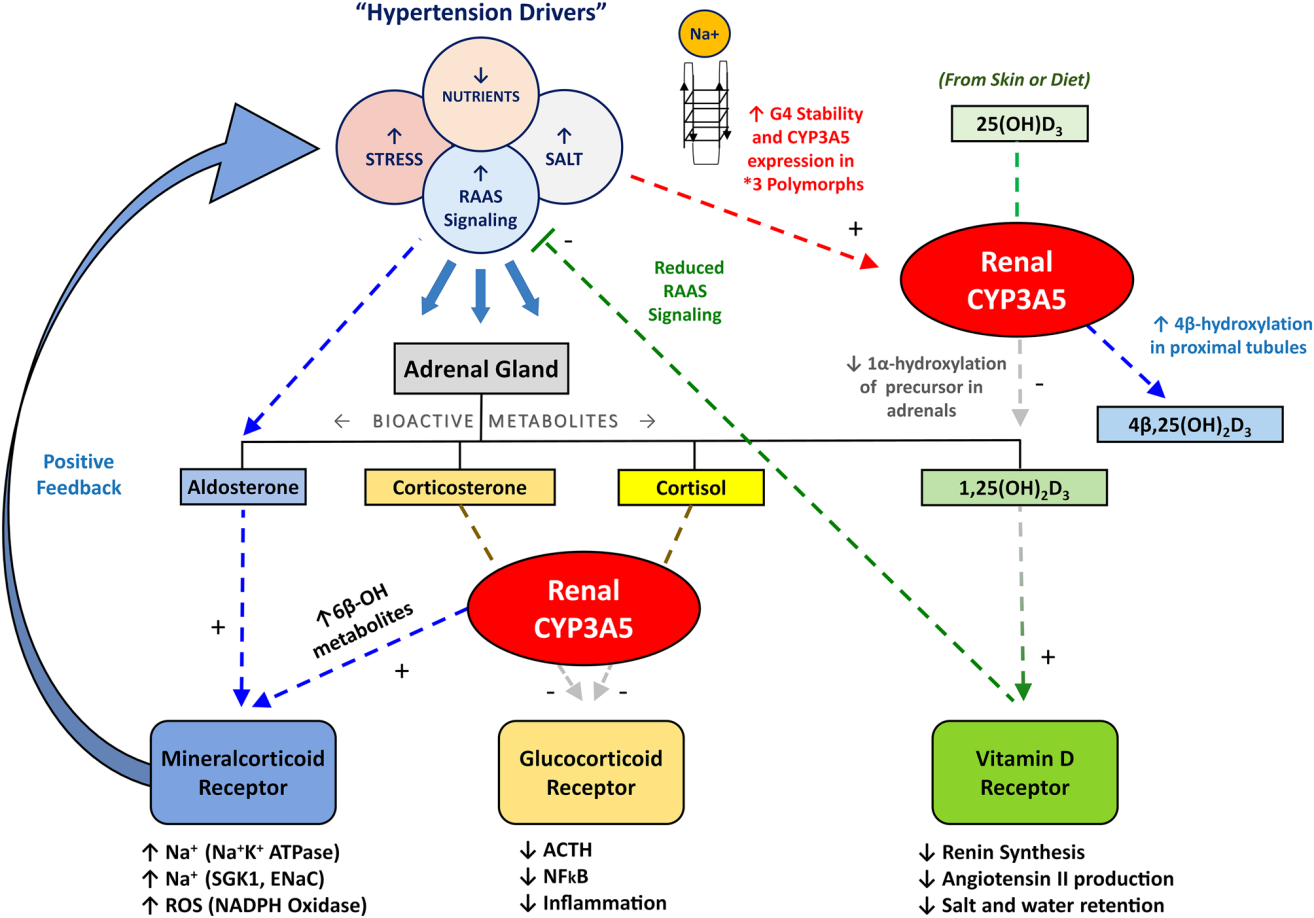
Schematic Diagram of CYP3A5′s Dual Role in the Regulation of Hypertension. Environmental factors (e.g., diet, chemical exposure, and stress) and homeostatic signaling mechanisms (i.e., Renin–Angiotensin–Aldosterone System (RAAS)) can alter renal and adrenal gland function to promote hypertension in humans. There is increasing evidence that renal *CYP3A5* activity plays a dual role in modulating vitamin D- and glucocorticoid-hormone signaling cascades that drive HBP pathology. First, CYP3A5 activity can convert stress-related glucocorticoids (corticosterone and cortisol) to mineralocorticoids via 6β-hydroxylation, which enhances MR signaling and triggers sodium retention (via several transporter related mechanisms) and increased ROS production. This activity also silences glucocorticoid signaling (via ACTH and NFkB) and increases inflammation. Second, *CYP3A5* can hijack endogenous vitamin D hormone signaling cascades by shunting 25-hydroxyvitamin D_3_ metabolism from CYP27B1 (1α-hydroxylase) towards a 4β-hydroxylation pathway that limits 1,25-dihydroxyvitamin D_3_ signaling via the VDR, reducing renin production that suppresses systemic RAAS activity. Thus, *CYP3A5* appears to play a pathological endogenous role in many human polymorphs by regulating positive feedback loops that drives the accumulation of stress hormones and sodium in the renal proximal tubule cells. Sodium cationic-stabilization of G4-like elements in CYP3A5 intron 3 may exacerbate this hypertensive signaling arc in CYP3A5 *1/*3 and *3/*3 polymorphs, who may conditionally express the gene. This salt-sensitive positive feedback loop may mask any adaptive effects of the 6986A/G polymorphism (rs776746) by promoting stress- and dietary-sensitive expression of CYP3A5, complicating any GWAS studies focused on the *1/*3 or *3/*3 genotypes.

These results are significant, because alterations in CYP3A5 expression levels can promote disease by disturbing the homeostasis of endogenous metabolites such as glucocorticoids, steroid hormones, and retinoids^3^. *CYP3A5*, unlike *CYP3A4*, is expressed in steroidogenic organs including the prostate, adrenals and kidney, making it a major mediator of pleiotropic, hormone signaling events^3^. The renal localization of *CYP3A5*, within the proximal tubule and collecting duct, allow for the local conversion of glucocorticoids including corticosterone to metabolic products like 6β-hydroxycorticosterone, which promote paracrine signaling and a metabolic switch from glucocorticoid to mineralocorticoid activity. Prolonged *MR* signaling leads to enhanced salt retention, which in turn, promotes hypertension (see Fig. 6).

The *CYP3A5* response to glucocorticoids requires dual activation of two, glucocorticoid response elements (GRE) in the promoter^94^. As mentioned above tonicity-responsive enhancer located in intron 2 of *CYP3A7* also acts as an enhancer for neighboring *CYP3A4* and *CYP3A5* expression, through interactions with NFAT5, a binding protein that augments salt-induced *CYP3A5* expression^80^. Transcriptional *CYP3A5* amplification from stress-induced release of glucocorticoids and salt intake, combined with salt retention, and vitamin D hormone insufficiency, can all contribute to hypertension via modulation of the renin–angiotensin–aldosterone-system (RAAS) (Fig. 6).

A high Na + /low K + diet also promotes hypertension and K + supplements can reduce blood pressure in hypertensive patients^95,96^. In contrast, aldosterone is normally secreted in response to hyperkalemia, and therefore the shunting of glucocorticoids to the *MR* pathway by *CYP3A5* during elevated stress could potentially disrupt innate K + feedback loops required to prevent hypokalemia. Human migration from equatorial regions that increased vitamin D insufficiency, and an increasingly high salt diet, may have reduced the fitness of the sodium sparing CYP3A5*1 genotype in some populations^97,98^. Today, the CYP3A5*3 allele is most commonly found in people of European ancestry, with allele frequencies ranging from 0.14 among sub-Saharan Africans to > 0.95 in European populations^98^. CYP3A5*3 polymorphs may have acquired an advantageous mutation in *CYP3A5* intron 3, that slowed renal metabolism of key hormones, and alleviated the tendency to excrete K + and retain Na + during stress. However, because the SNP regulating CYP3A5*3 variant expression is located near a putative G4 structure that responds to fluctuations in intracellular K + levels, it appears to represent a conditional mutation, rather than an obligate null mutation.

It is notable that several human GWAS studies have failed to link any *CYP3A5* polymorphisms to hypertension^10,18,19^. This is not surprising as the confounding potential of the environment to modulate CYP3A5*3 gene expression explains why penetration of the SNP phenotype is incomplete. We conclude that a G4-like structure within the *CYP3A5* intron 3 forms a cation-sensitive “switch” that can suppress NMD in CYP3A5*3 polymorphs. This is the first report of a transient restoration of CYP3A5*3/*3 expression in vitro*,* potentially mediated by a G4 pre-mRNA element.

While our results suggest that gene-directed approaches targeting *CYP3A5* for the treatment of HBP are viable and deserve greater consideration, ASO-induced nephrotoxicity is a hard-to-predict adverse event that may only be discovered once the molecule enters clinical trials. Consequently, new models and biomarkers need to be tested for their ability to screen for renal liabilities earlier in drug discovery to facilitate the development of safer ASOs. As backbone chemistry may be a core determinant of the safety of ASOs toward renal cells, we utilized a PT-MPS to characterize the safety of a PMO and two 2′-OMe-PSOs. KIM-1 secretion was not affected by treatment with HPV PSO or DSP PSO while being slightly reduced by HPV PMO. A previous report showed KIM-1 secretion by renal tubule cells in vitro in response to toxic locked-nucleic acid modified gapmers was sequence-dependent and variable^99^. Additional compounds, particularly those with sequences known to be toxic, should be tested to fully evaluate the value of KIM-1 secretion as a marker of ASO-induced injury in the PT-MPS. Likewise, secretion of miR-30e-5p needs to be evaluated with a larger matrix of 2′-OMe-PSO sequences to better determine its relationship to ASO-induced cell injury.

We also observed increased expression of genes involved in nucleoprotein activity based on ASO linkage chemistry, validating its role as a key driver of the intracellular distribution kinetics and protein binding properties of oligonucleotides. PSOs are known to associate with various RNA binding proteins to form nucleoprotein complexes^100–102^. Interaction of PSOs with these ribonucleoproteins can result in displacement of endogenous RNA molecules (e.g. NEAT1 from paraspeckles)^100^. Interestingly, among the proteins known to bind PSOs are nucleolin (NCL1) and nucleophosmin (NPM1), both of which have roles in regulating ribosome biogenesis and function^103–106^. NCL1 forms a complex with the U3 small nucleolar ribonucleoprotein (which contains SNORD3 RNAs) during the initial steps of small subunit processome formation^107^. Taken together, we postulate that the transcriptional response in DSP PSO treated cells may be driven by competition with endogenous molecules for nucleoprotein binding. Interestingly, toxic but not non-toxic phosphorothioate gapmers delocalize RNA binding proteins from the nucleolus in an RNAse H1-dependent manner, resulting in nucleolar stress and cell death^108^. While we did not observe overt cellular injury with DSP PSO treatment, the induction of genes involved in ribosome biogenesis warrants further investigation as it is an energetically demanding process.

Unlike the DSP PSO, we observed no changes in miRNA secretion or transcriptional signature with HPV PMO treatment, which strongly supports the safety of PMOs toward PTECs. However, further testing in in vivo may be required to differentiate the therapeutic value of these interpretations and we should not that differences in the metabolic stability of PSOs and PMOs in the PT-MPS model may not fairly predict their bioavailability in vivo*.* The inert nature of the HPV PMO is unlikely to be due to a lack of cellular entry, as activity was demonstrated in monolayer PTECs with gymnotic delivery of the 3A5*3 PMO (Fig. 1d). Thus, the PMO chemistry is an optimal choice for targeting renal CYP3A5 as it exhibits an excellent safety profile with adequate pharmacology*.*

## Methods

### Reagents

DMEM (Dulbecco's Modified Eagle Medium) media and additives were obtained from Sigma (St. Louis, MO). Fetal Bovine Serum was obtained from Atlanta Biologics (Flowery Branch, GA). TMPyP4 (sc-204346) was obtained from Santa Cruz Biotech (Dallas, TX). Gentest 7-benzyloxy-4-[trifluoromethyl]-coumarin (BFC; #451730) and 7-hydroxy-4-[trifluoromethyl]-coumarin (HFC; #451731) were obtained from Corning (Corning, NY). Cortisol (H-0888) and 6β-hydroxycortisol (H-6904) were obtained from Sigma (St. Louis, MO).

### Cell culture

Human embryonic kidney proximal tubule epithelial cells (HEK293), and African green monkey kidney cells (CV-1), were purchased from ATCC.org (Manassas, VA). Both cell lines were grown DMEM media plus 10% Fetal Bovine Serum and 2 mM L-Glutamine (Corning; #25-005-CI) supplemented with 1X Pen-Strep (Gibco; #15140–122). Sanger DNA sequencing was used to validate the proper sequence of CYP3A5 gene in both cell lines (CGRB services, Oregon State University). Experimental and Scrambled Control PMOs used in cell culture experiments (0.1 – 5 mM) were administered using passive uptake.

Primary human PTECs were sourced from kidney tissue obtained from patient nephrectomies at the University of Washington Medical Center in collaboration with Northwest BioTrust, as described previously^71^. Human PTECs were isolated as previously described (Weber et al. 2016), in accordance with a protocol approved by the University of Washington Human Subjects Institutional Review Board (IRB STUDY00001297)^71^. This Human Subjects study qualified for expedited review (“minimal risk”; Category 5). The kidney tissue is considered anonymized surgical waste with no patient identifying factors collected. Informed consent was obtained from all tissue donors. Cells were maintained as described previously with the exception of donor 2, which was maintained in DMEM/F12 No Glucose (US Biological, D9807-02) supplemented with 5 mM Glucose (Sigma, G7528), 14.3 mM Sodium bicarbonate (Sigma, S-6297), ITS (sigma, I-1884-1VL), 25 ng/mL Prostaglandin E1 (Sigma, P5515), 25 ng/mL Hydrocortisone (Sigma, H-0135), 3.5 ug/mL L-Ascorbic acid (Sigma, A-4544), 3.652 ng/mL Sodium selenite (Sigma, S-9133), 5 pM 3,3′,5-Triiodo-L-thyronine sodium salt (Sigma, T-5516), 10 ng/mL rhEGF (R&D Systems, 236-EG-200), and Antibiotic–Antimycotic (Gibco, 15240062). See Supplemental Table 1 for donor demographics.

### Nucleic acid and antisense oligomer synthesis

The 8 antisense oligomers used in this study, and their targets, are listed in Table 1 along with their anticipated biological significance. The oligomers were synthesized as phosphorodiamidate morpholino oligomers (PMOs or morpholinos) by Gene-Tools, LLC (Philomath, OR, (http://www.gene-tools.com/history_production_and_properties#preparation) as described earlier by Summerton and Weller^109^. Every PMO generated by Gene-Tools is accompanied by an oligomer properties specification sheet and a spectral analysis of the product to demonstrate removal of waste products by selective precipitation (synthesis resin, ammonia, cleaved base-protective groups, and minor amounts of short truncation fragments).

The 5 DNA oligomer sequences used in gel mobility studies are listed in Table 2. DNA and RNA oligomers were purchased from Integrated DNA Technologies (Coralville, IA).

### Nucleic acid gel mobility shift assay

Nucleic acid mobility shift assays were performed using the method of Nambiar et al.^110^ Oligomers, including *HOX11* C- and G-strand controls (not shown), were incubated in 10 to 100 mM KCl in Tris–EDTA buffer pH 8.0 at 37 °C for 1 h. The different G4 structures were resolved on 12% native polyacrylamide gels in the presence of 10 mM KCl in both gel and the running buffer, at 150 V at 4 °C. The gels were stained with ethidium bromide and photographed on a UV-transilluminator. Original uncropped and unmodified gel images for DNA and RNA gel shifts shown in Fig. 3b,c are presented in Supplemental Fig. 4.

### RNA isolation and PCR analysis

Total RNA was isolated from HEK293 or CV-1 cells using the QIAshredder (Qiagen; #79654) and RNEAsy Mini Kit (Qiagen; #74104). RNA was treated with DnaseI (New England Biolabs; 1 U/ µg RNA; 30 min at 37 °C; 5 min at 75 °C) to remove genomic DNA prior to reverse transcription of mRNA to cDNA using the iScript cDNA synthesis kit (Bio Rad; #170–8890). Endpoint PCR was performed using the Platinum Taq High Fidelity DNA Polymerase kit (Invitrogen; #11,304–011) using the manufacturer’s recommended protocol with minor modifications; optimal annealing temperatures for the *CYP3A5* primer sets were determined empirically, between 50 and 55 °C for 30 s. PCR products were visualized on 1% UltraPure Agarose gels (Invitrogen; #15510-027) prepared in 1X TBE buffer and stained with Ethidium Bromide Solution (Calbiochem; #4410). Exon 4 inclusion in *CYP3A5* mRNA transcripts was monitored using the following primer sets: hCYP3A5_dex4_FP1-5′-GGA AAC CTG GCT TCT CCT G-3′ and hCYP3A5_dex4_RP1-5′-TGA CAG GCT TGC CTT TCT CT-3′. All primers were purchased from IDTDNA.com (Coralville, IA).

qRT-PCR analysis was performed using the iQ SYBR Green Supermix (BioRad; #170–8880) in the ABI PRISM 7500 FAST Sequence Detection System (Applied Biosystems; 2 h run protocol; 96-well plate) using the manufacturer’s recommended settings for SYBR Green qPCR analysis. Assays were performed in technical quadruplicates using pre-validated, Primetime qPCR primer set assays from for human target genes: *CYP3A4* (Hs.PT.58.19392980); *CYP3A5* (Hs.PT.58.41063109), *18 s RNA* (Hs.PT.39a.22214856.g) and *ACTB* (Hs.PT.39a.22214847), all obtained from Integrated DNA Technologies (Coralville, IA). Template (10–25 ng) and primer concentrations (0.5-1X Primetime mix) were optimized for each primer set to eliminate primer dimer formation in final experimental conditions. Results of 4 replicate biological samples were quantified using the 2(-Delta C(T)) method and relative fold changes were normalized to the relative expression of 18S RNA in each sample^81^. ACTB was chosen as an alternative internal standard but was not used to calculate the results. Standard deviation (SD) was calculated using replicate delta C_T_ values, as described^80^. Microsoft Excel and GraphPad Prism were used for statistical evaluation of data, and statistical comparisons were made using the Student t-test among individual delta C_T_ replicates. Statistical significance was considered at *p* values < 0.05.

### Western immunoblotting

Total protein was extracted from HEK293 and CV-1 cell lines using the RIPA Buffer Lysis System (Santa Cruz Biotech; #sc-24948). Fresh RIPA buffer was prepared just prior to extraction and supplemented with protease inhibitors (Complete Mini; Roche; #11777500; 1 tablet/10 mL RIPA buffer). After removing cell culture media, cells were washed with 1 × PBS (VWR), and removed from plates with a rubber policeman in 1 mL of cold 1X PBS. Cells were pelleted at 3,000 rpm for 5 min. Pellets were re-suspended in 0.75 mL of fresh RIPA buffer and incubated on ice for 30 min with intermittent vortexing. Supernatant was then separated from cell debris via centrifugation at 14,000 × g for 15 min at 4 °C. Samples were then frozen at − 80 °C or prepared for Western Blot analysis immediately via suspension into 4X Laemmli Sample Buffer (BioRad; #161-0747) supplemented with 400 mM β-mercaptoethanol (Sigma; #M6250). Western blot analysis was completed using the BioRad Mini-PROTEAN Electrophoresis System (Hercules, CA). Standard manufacturer settings and protocols were used for blotting, with minor modifications, including alternate sample preparation procedures that included the storage of proteins in fresh 1X Laemmli Sample buffer at 4 °C, overnight, prior to boiling) to facilitate denaturation of hydrophobic target proteins. Monoclonal antibodies for human *CYP3A5* ((F18 P3 B6) were obtained from ThermoFisher Scientific (Waltham, MA; Cat# MA3-033; RRID:AB_2090513; Ref: PMID:15897573) and used at 1–2 µg/ml. Monoclonal antibodies for *CYP3A4* (HL3) were obtained from Santa Cruz Biotechnology, Inc. (Dallas, TX; Cat# sc-53850; RRID:AB_782375; Ref: PMID: # 27732883) and used at dilutions between 1:100 and 1:200. Monoclonal antibodies for the housekeeping gene *GAPDH* (G-9) were obtained from Santa Cruz Biotechnology, Inc. (Dallas, TX; Cat# sc-365062; RRID:AB_10847862; Ref: PMID: 28977600) and were used at a dilution of 1:100. Total and variant CYP3A5 protein expression was determined quantitatively using grayscale, densiometric analysis in ImageJ^111^. Original uncropped images for blots shown in Fig. 4a are presented in Supplemental Figs. 5–7.

### CYP3A5 enzyme activity assays

HEK293 cells were incubated with substrate 7-benzyloxy-4-[trifluoromethyl]-coumarin (BFC) which is converted into product 7-hydroxy-4-[trifluoromethyl]-coumarin (HFC)^112^. The HFC was detected at excitation wavelength of 409 nm and emission wavelength of 530 nm. All readings were compared to a standard curve with HFC. HEK293 cells were grown in 35 mm dishes in media supplemented with 1 nM cortisol.

An LC MS/MS method was developed for simultaneous evaluation of cortisol (CORT) and 6β-hydroxycortisol (6βOH) levels on an MDS Sciex 4000 Q TRAP LC–MS/MS System (Applied Biosystems). CORT (363 → 121) and 6βOH (379.3 → 343.1) fragments were monitored and standard curves were prepared for each fragment. Control and conditioned media samples (N = 4) were extracted in methylene chloride and then resuspended in 10% methanol for AUC analysis. Targeted metabolomic analysis was conducted using a Poroshell 120 EC-C18 column (Agilent; 2.1 × 50 mm, 2.7 mm) and a standard water:ethanol gradient (10–95%) containing 5% formic acid. Standard curves (n = 3) for quantifying fragment ion peaks are shown in Supplemental Fig. 8.

### Mineralocorticoid receptor activity

The hypothesis that *CYP3A5* activity will transform glucocorticoids, cortisol, and corticosterone, into 6β-hydroxy metabolites gaining mineralocorticoid activity was evaluated using the Human MR Reporter Assay System (#IB00501-32) from Indigo Biosciences (State Park, PA). The media from cells incubated in control media and media supplemented with 12 mM KCl (24 h; N = 4) were added to a cell-based reporter system monitoring mineralocorticoid receptor (MR) transactivation as described in the manufacturer’s protocol.

### CYP3A5 oligonucleotide treatment in PTEC cells

PTECs passage 2–4 were seeded into uncoated 24-well plates at 50,000 cells/well and allowed to recover for 24 h before treatment with PMO for 6 days. Fresh treatment media was added after 72 h. On the 7th day, the cells were cotreated with PMO and 1 µM Midazolam (Sigma, M-908) for 24 h.

### Midazolam extraction and quantification

At the end of treatment, the supernatant was collected, and the wells were washed with 500 µL methanol containing 40 ng/mL 1-OH-MDZ-d4 (Sigma, H-921) followed by another 500 µL methanol wash and addition of 1 mL methanol to each sample. The samples were stored at -80 degrees Celsius until extraction. Standards were prepared fresh daily by spiking 1-OH-midazolam (0.025–3.2 ng; Sigma, H-902) and 1-OH-MDZ-d4 (20 ng) into PTEC maintenance media. The amount of 1-OH-MDZ produced by PTECs was quantified by LC/MS–MS, as described previously with minor modification^113^. Briefly, the extracts were reconstituted in a 50:50 mixture of water:methanol and analytes were separated using the following gradient conditions: solvent A (0.1% formic acid in water) and solvent B (100% methanol) started at 50% for the first minute, solvent B increased linearly to 90% from 1 to 3 min, was held at 90% until 6 min, then returned to 50% from 6.1 to 10 min. Mobile phases were pumped at a flow rate of 0.25 mL/min.

### Generation and treatment of PT-MPS

Microfluidic devices were purchased from Nortis Biotechnology (Woodinville, WA) and PTEC tubules were generated as reported previously^71,73^. All tubules were allowed to culture for 5–7 days before initiating treatment. Oligonucleotide stocks were prepared to 1 mM with water and stored at room temperature. Working solutions were prepared in PTEC maintenance media. All treatments were administered to devices via syringe-pump-driven perfusion (KD Scientific Inc., model KDS220) at a flow rate of 0.5 µL/min.

### HO-1 immunocytochemistry and quantification

All procedures were performed at room temperature and all solutions were perfused through the devices at a flow rate of 10 µL/min. The tubules were fixed with Formalin, Buffered, 10% (Fisher, SF100-4) for 30 min, followed by a wash with DPBS (ThermoFisher, 14040133) for 60 min. The devices were stored at 4 °C until further processing. To label HO-1, the tubules were first blocked and permeabilized with dPBS containing 5% bovine serum albumin (BSA) and 0.1% Triton X-100 for 2 h. Rabbit monoclonal anti HO-1 primary antibody (Abcam, ab52947) was diluted 1:100 in DPBS containing 5% BSA and perfused for 1 h, followed by a wash with DPBS containing 0.05% tween-20 for 2 h. Goat anti rabbit secondary (Fisher, A11037) was diluted 1:1000 in DPBS + 5%BSA and perfused for 1 h followed by a 2-h wash with DPBS + 0.05%Tween 20. Nuclei were labeled with DAPI and imaged on a Nikon Eclipse Ti-S microscope. The fluorescence intensity of HO-1 labeling was quantified using ImageJ software.

### KIM-1 ELISA and chemiluminescent assay

Levels of KIM-1 protein were quantified from 50 µL or 100 µL of device effluents according to the manufacturer’s instructions using a chemiluminescent assay (Meso Scale Discovery, K151JHD-2) or ELISA (R&D Systems, DY1750B), respectively.

### miRNA analysis

Effluent was collected over the 44 h and 200uL was used for qPCR analysis as previously described^73^.

### RNA extraction and sequencing

PTECs were harvested from devices by injecting 100 µL of detergent (Abcam luminescent kit, part 8206000) into the injection port using a 1 mL slip-tip syringe (BD, 309659) equipped with a 22-gauge needle (BD, 305142). Cell lysate was collected into 900 µL Trizol and frozen at -80 degrees Celsius until extraction. RNA was isolated using a RNeasy Micro Kit (Qiagen, 74004) and the RNA library was prepared and sequenced as described previously^73^. Aligned data were read into R and summarized as counts per gene using the Bioconductor TxDb.Hsapiens.UCSC.hg38.knownGene package in concert with the GenomicAlignments package (version 3.4.0).

### Statistical analysis

Data are expressed as mean and standard deviations were derived from replicate samples (at least three per treatment group). Statistical analyses were performed using GraphPad Student’s two-tailed *t*-test and ANOVA with Tukey–Kramer multiple comparisons test. Differences were considered significant at *p* < 0.05. Statistical analysis for HO-1, KIM-1 and miRNA analysis was carried out in R (version 4.0.0) using the lm function, and the Anova function from the car package was used to compute two-way or 3-way ANOVA test (Type-III sums of squares). The glht function from the multcomp (version 1.4) package was used for the Tukey’s multiple testing comparisons.

Statistical analysis for global transcriptomics was carried out using R (version 3.4.0). Before fitting any models, we first excluded any genes that were expressed at consistently low levels across all samples. Prior to filtering, we had 25221 genes and after filtering, we have data for 13779 genes. We then performed a trimmed mean of M-values (TMM) normalization^114^. To test the treatment difference, we used a GLM framework with quasi-likelihood method in edgeR package (v3.18.1)^115^. Rather than using a post hoc fold change filtering criterion, we used the glmTreat function from edgeR, which is analogous to the TREAT approach^116^. We selected genes based on a threshold of 1.1-fold-change and a false discovery rate of 10%.

## Supplementary Information

Supplementary Information
