# Author Correction: Antisense oligonucleotide development for the selective modulation of CYP3A5 in renal disease

**DOI:** 10.1038/s41598-021-90075-z

**Published:** 2021-05-17

**Authors:** Kevin A. Lidberg, Andrew J. Annalora, Marija Jozic, Daniel J. Elson, Lu Wang, Theo K. Bammler, Susanne Ramm, Maria Beatriz Monteiro, Jonathan Himmelfarb, Craig B. Marcus, Patrick L. Iversen, Edward J. Kelly

**Affiliations:** 1 Department of Pharmaceutics, University of Washington, Seattle, WA USA; 2 Department of Environmental and Molecular Toxicology, Oregon State University, Corvallis, OR USA; 3 Department of Environmental and Occupational Health Sciences, University of Washington, Seattle, WA USA; 4 Victorian Centre for Functional Genomics, Peter MacCallum Cancer Centre, Melbourne, Australia; 5 Depto Clinica Medica, Faculdade de Medicina FMUSP, Universidade de Sao Paulo, São Paulo, Brazil; 6 Kidney Research Institute, University of Washington, Seattle, WA USA

Correction to: *Scientific Reports* 10.1038/s41598-021-84194-w, published online 25 February 2021

This Article contains an error where the Acknowledgements section is incomplete.

“The authors wish to thank the Oregon State University Agricultural Research Foundation and LS Pharma for financial and material support for this project. We also wish to thank the College of Agricultural Sciences at Oregon State University, the OSU Center for Genome Research and Biocomputing, and the OSU Mass Spectrometry Center for access to their infrastructure and facilities as needed to conduct these studies. We would also like to thank the University of Washington PK Laboratory for mass spectrometry support. Research reported in this publication was supported in part by the National Institutes of Health National Center for Advancing Translational Sciences awards UG3TR002158, UH3TR002158 & UG3TR002178, National Institute of Environmental Health Sciences Interdisciplinary Center for Exposures, Diseases, Genes and Environment (UW EDGE Center; P30ES007033)), T32 (EP&T training grant—ES007032 – 41A1) (KAL) and the generous unrestricted gift from the Northwest Kidney Centers to the Kidney Research Institute.”

should read:

“The authors wish to thank the Oregon State University Agricultural Research Foundation and LS Pharma for financial and material support for this project. We also wish to thank the College of Agricultural Sciences at Oregon State University, the OSU Center for Genome Research and Biocomputing, and the OSU Mass Spectrometry Center for access to their infrastructure and facilities as needed to conduct these studies. We would also like to thank the University of Washington PK Laboratory for mass spectrometry support. Research reported in this publication was supported in part by the National Institutes of Health National Center for Advancing Translational Sciences awards UG3TR002158, UH3TR002158 & UG3TR002178, National Institute of Environmental Health Sciences Interdisciplinary Center for Exposures, Diseases, Genes and Environment (UW EDGE Center; P30ES007033)), T32 (EP&T training grant—ES007032 – 41A1) (KAL) and the generous unrestricted gift from the Northwest Kidney Centers to the Kidney Research Institute. A portion of this work was supported by T32 (EHS training grant—ES007060) (DJE).”

