# Supplementary material for: Antisense oligonucleotide development for the selective modulation of CYP3A5 in renal disease
Source: Sci Rep. 2021 Feb 25;11:4722. doi: 10.1038/s41598-021-84194-w (PMC7907328; doi:10.1038/s41598-021-84194-w)
Supplement: Supplementary file 1 — Supplementary Information [file 41598_2021_84194_MOESM1_ESM.pdf]

SUPPLEMENTAL MATERIALS

Antisense Oligonucleotide Development for the Selective Modulation of CYP3A5 in Renal Disease.

Kevin A. Lidberg[1¶], Andrew J. Annalora[2¶*], Marija Jozic[2], Daniel J. Elson[2], Lu Wang[3], Theo K. Bammler[3], Susanne Ramm[4], Maria Beatriz Monteiro [5], Jonathan Himmelfarb[6], Craig B. Marcus[2], Patrick L. Iversen[2], and Edward J. Kelly[1*]

[1]Department of Pharmaceutics, University of Washington, Seattle, Washington, USA

[2]Department of Environmental & Molecular Toxicology, Oregon State University, Corvallis, Oregon, USA

[3]Department of Environmental and Occupational Health Sciences, University of Washington, Seattle, Washington, USA

[4]Victorian Centre for Functional Genomics, Peter MacCallum Cancer Centre, Melbourne, Australia

[5]Depto Clinica Medica, Faculdade de Medicina FMUSP, Universidade de Sao Paulo, São Paulo, Brazil

[6]Kidney Research Institute, University of Washington, Seattle, Washington, USA

***** Corresponding Authors

E-mail: Andrew Annalora - Andrew.Annalora@oregonstate.edu

E-mail: Edward Kelly - Edkelly@uw.edu

¶ Authors contributed equally to this work.

Supplemental Figures

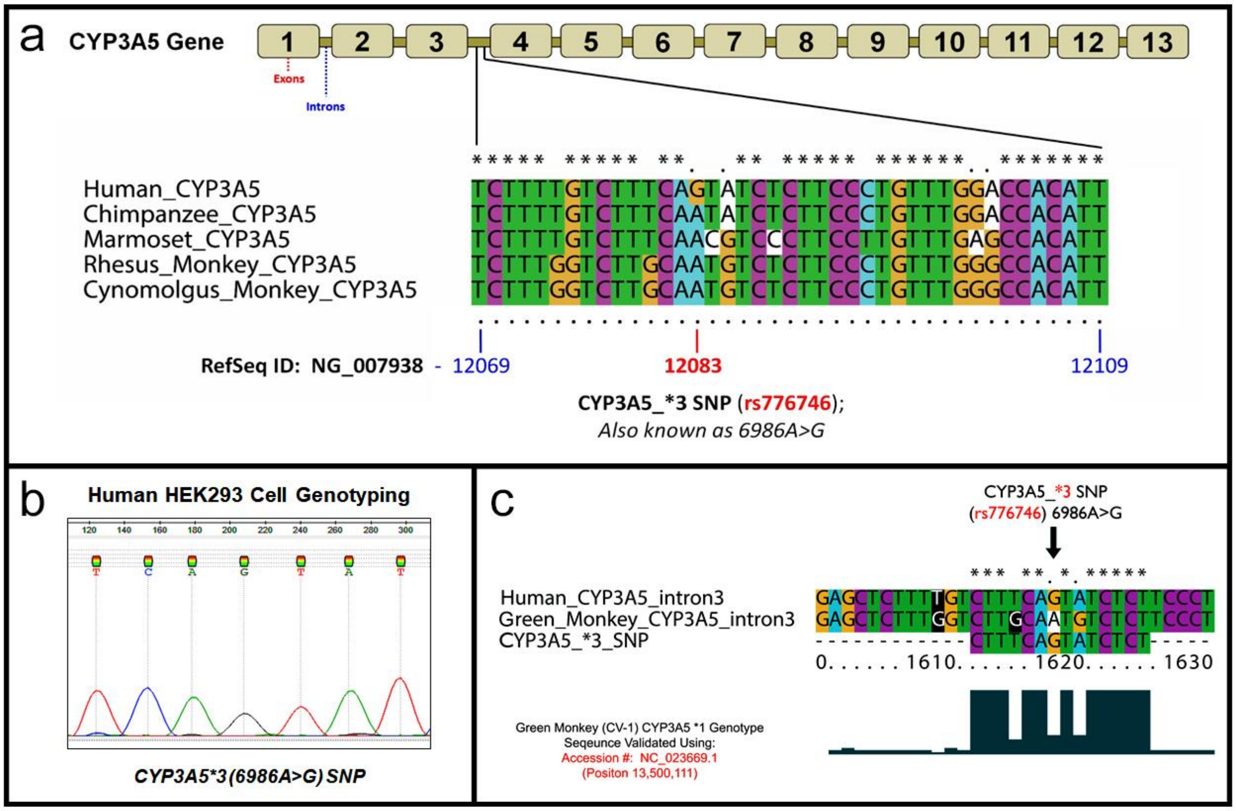

Supplemental Figure 1. Multiple Sequence Alignment of the Intron 3 region of *CYP3A5* and Genomic Sequencing of the *CYP3A5* gene in HEK293 and CV-1 cells lines a.) DNA sequence alignment comparisons among human and various nonhuman primate DNA sequences reveal consensus within human intron 3 region near the *CYP3A5*3* (*6986A>G)* SNP site. The reference human sequence (*NG_007938.1*) reveals the presence of the *CYP3A5*3* SNP at position 12083. All other primate sequences, including chimpanzee (*NC_006474.4*), marmoset (*NC_013897.1*), rhesus monkey (*NC_027895.1*) and cynomolgus monkey (*NC_022274.1*) express the *CYP3A5*1* SNP at this site. The *6986A>G* mutation creates a cryptic splice acceptor site in intron 3 that can create alternatively spliced transcripts subject to NMD). **b.**) The *CYP3A5* intron 3 sequence region from HEK293 cells indicates they are **3/*3* genotype (right). **c.**) Genomic Sequencing (Accession #: NC_023669.1) confirms that CV-1 cells express the CYP3A5*1 transcript like other non-human primates, shown in Supplemental Figure 1a.

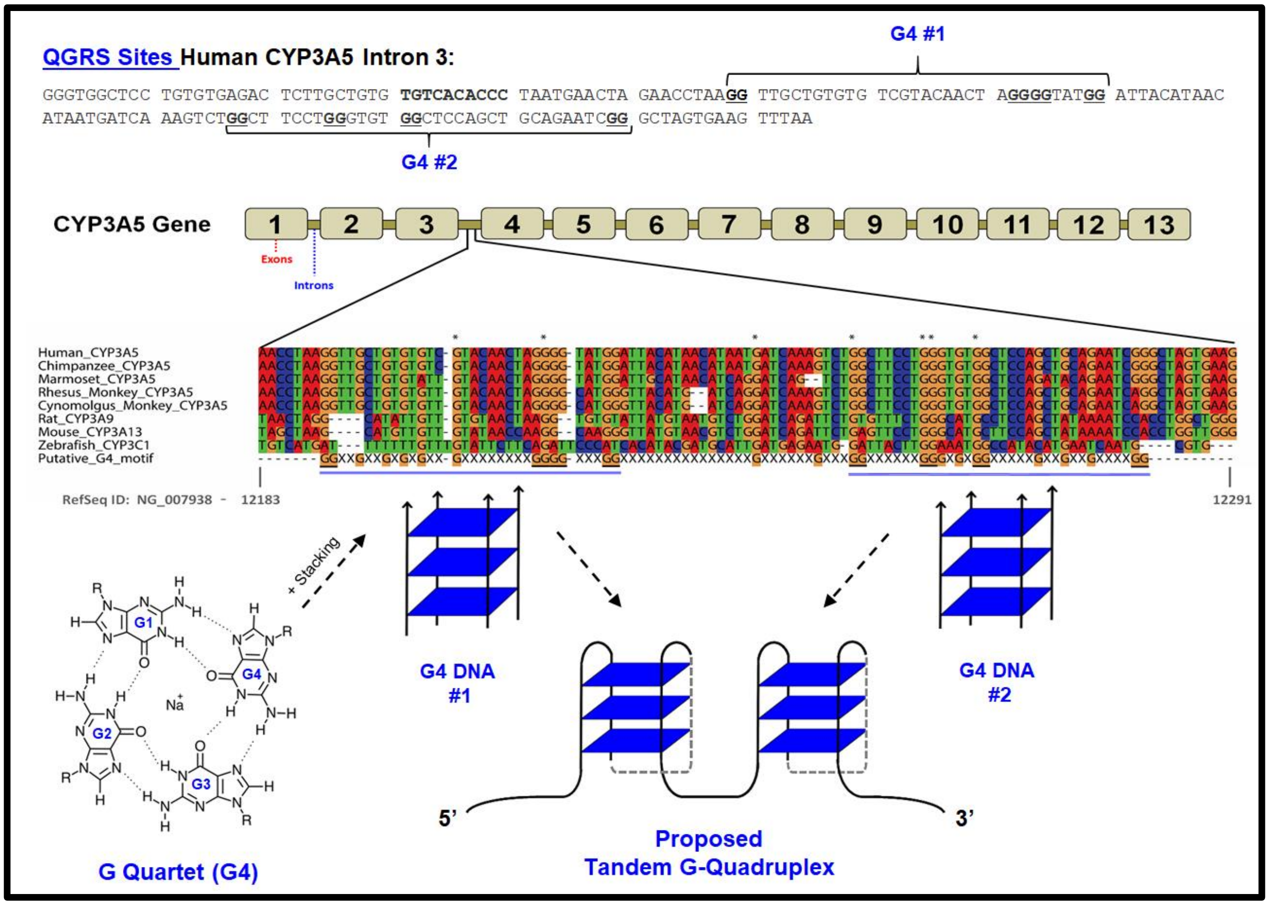

Supplemental Figure 2. Computational Analysis of CYP3A5 Intron 3 Reveals a Putative G Quadruplex Structure in Proximity to the CYP3A5*3 SNP site. a.) The program QGRS-Conserve was used to identify two putative G quadruplex (G4) sequences in the intron 3 region of the human *CYP3A5* gene; the two putative G quartet (G4) motifs are shown. **b.**) DNA sequence alignment analysis of the intron 3 region of the *CYP3A5* gene (or the species-specific ortholog) among humans (*NG_007938.1*), various nonhuman primates (chimpanzee (*NC_006474.4*), marmoset (*NC_013897.1*), rhesus monkey (*NC_027895.1*), cynomolgus monkey (*NC_022274.1*), rodents (rat (*NC_005111.4*), mouse (*NC_000071.6*) and fish (zebrafish *NC_007114.7*)) reveal strong consensus in the intron 3 region located between the *CYP3A5*3* (*6986A>G; rs776746*) SNP site and intron 3/exon 4 splice junction. A larger G quartet structure (151 base pairs) overlapping the two smaller G4 motifs, not identified by the program, was

identified visually (not shown). **c.**) Schematic representation of the G quartet and G4 structure; four guanine Hoogstein base pairing, fundamental to G4 structures is shown with an internal, stabilizing potassium ion (K+). The proposed assembly of a tandem G quadruplex structure in the region adjacent to *CYP3A5*'s intron 3/exon 4 splice junction is also depicted. G-quadruplex (G4) structure images used in this figure were adapted from the original art "G-quadruplex (G4) structure" by Harris LM and Merrick CJ, which is licensed under CC-BY-4.0.

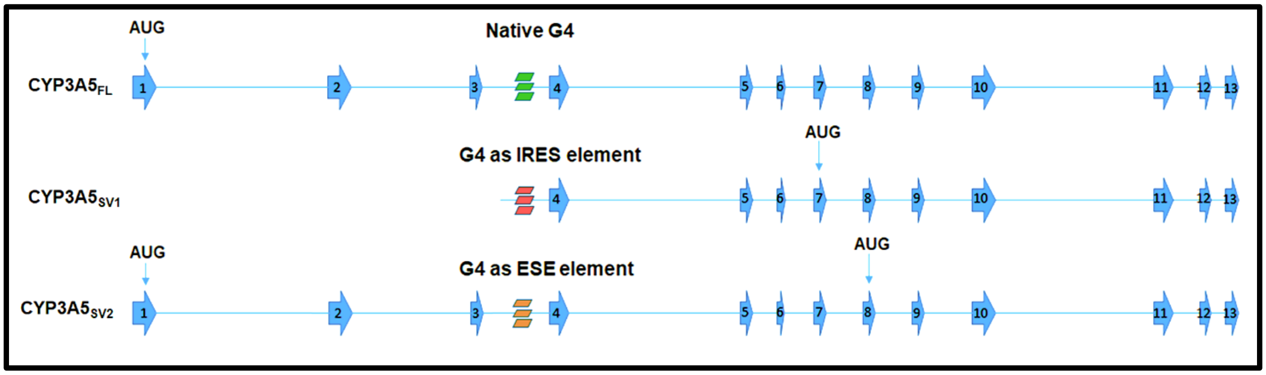

Supplemental Figure 3. Models for G4-mediated-induction of *CYP3A5* splice variant proteins in CYP3A5*3/*3 HEK293 cells. We detected a prominent 30 kDa splice variant of *CYP3A5* in HEK293 cells treated with potassium or sodium cations. We propose the 151bp G4 motif in intron 3 serves as either an internal ribosomal entry site (IRES) or an exonic splicing enhancer (ESE) element redirecting translational initiation to start sites in exons 7 or 8, or the skipping of multiple internal exons, which remain uncharacterized.

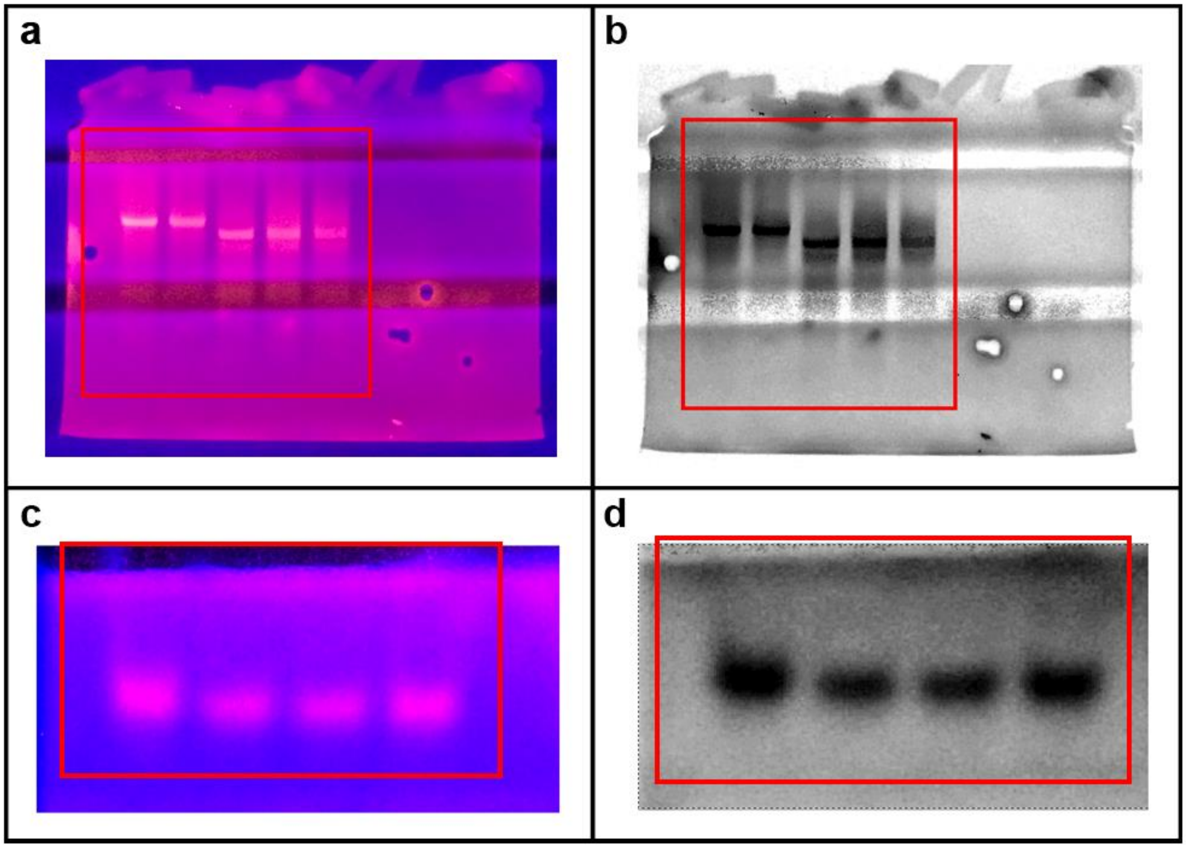

Supplemental Figure 4. Unprocessed RNA/DNA Gels Examining G4-like Structures in the CYP3A5 intron 3. **a.)** An uncropped and unmodified gel image from the DNA gel shift mobility assay highlighted in Figure 3b is shown. **b**.) This image was converted to grayscale and contrast enhanced for analysis using Adobe Photoshop. Cropped sections of the DNA blot shown in Figure 3b are highlighted in the red boxes in panels **a**.) and **b**.). **c**.) An uncropped and unmodified gel image from the RNA gel shift mobility assay highlighted in Figure 3c is shown. **d.)** The original image was converted to grayscale and contrast enhanced for analysis using Adobe Photoshop. Cropped sections of the RNA blot shown in Figure 3c are highlighted in the red boxes in panels **c**.) and **d**.).

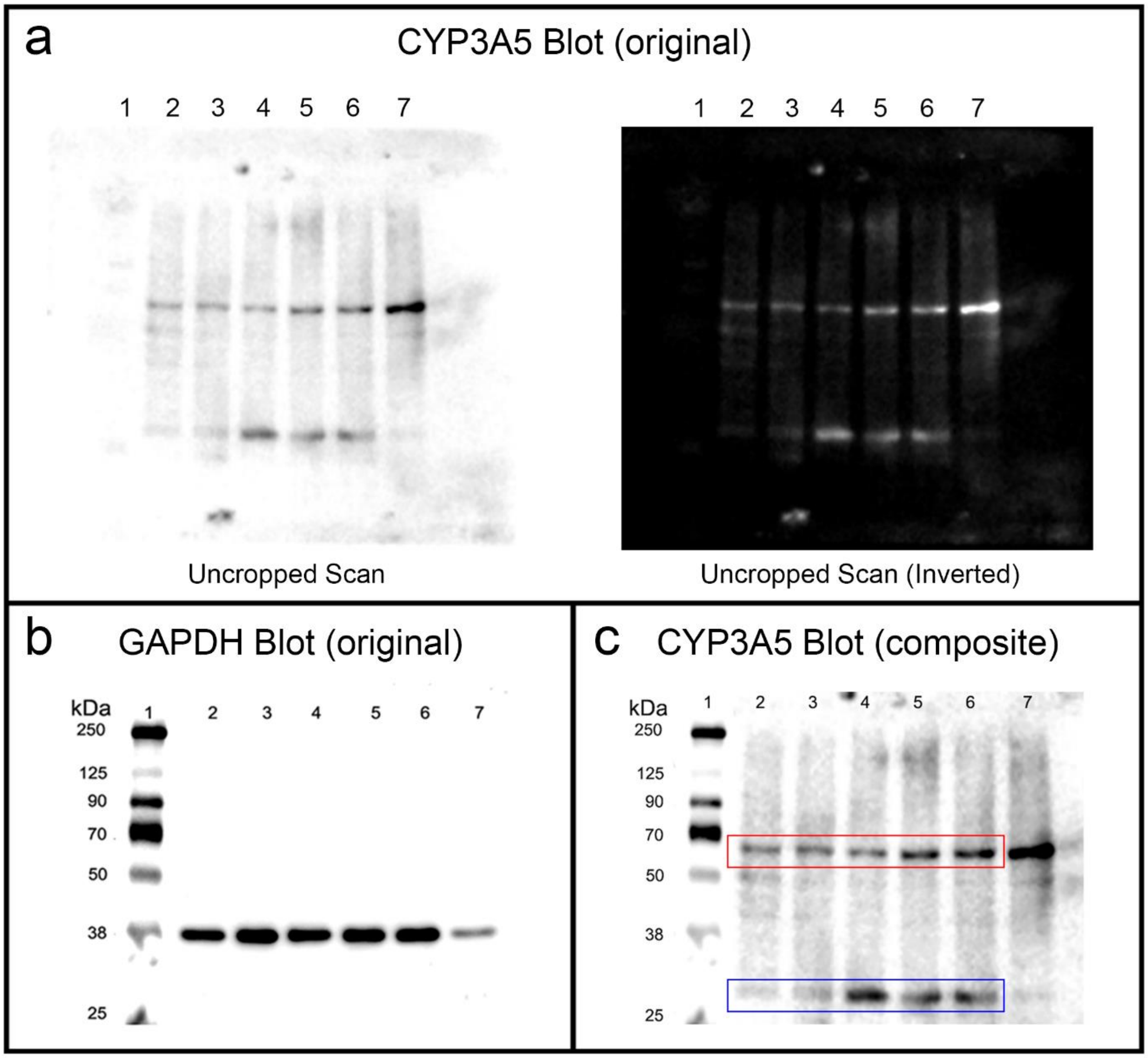

Supplemental Figure 5. Uncropped Western Blots for CYP3A5 and GAPDH expression in HEK293 cells. **a.**) Western blot data shown in Fig. 4a was cropped from the original CYP3A5 blot shown in panel a. Blot images were captured using the Azure Biosystems c600 imager and original unmodified auto scans (normal and inverted) are shown. **b.**) The blot membrane was initially probed with monoclonal antibodies for CYP3A4 (not shown), and then stripped (with Restore Plus Western Blot Stripping Buffer; ThermoScientific #46430) prior to re-probing with the CYP3A5 and GAPDH antibodies. After stripping the CYP3A4 antibody, the molecular weight

marker was poorly resolved when re-probing with the CYP3A5 and GAPDH antibodies, but the composition of the CYP3A5 blot could still be oriented using weakly visible bands at 90, 70 and 25 kilodaltons (kDa). **c.**) To aid in interpretation, a composite version of the CYP3A5 blot was developed with the molecular weight marker from the original CYP3A4 blot superimposed onto the marker bands of the CYP3A5 blot. Cropped sections of the CYP3A5 blot shown in Figure 4a are highlighted in the red box ($CYP3A5_{WT}$) and blue box ($CYP3A5_{SV}$) and were matched with corresponding lanes in the GAPDH blot (lanes 2-6). Uncropped and unmodified versions of both the CYP3A5 and GAPDH blots are shown in Supplemental Figures 6 and 7, respectively. Quantitative analysis using ImageJ was performed on cropped sections of the original blot, which were contrast enhanced in Adobe Photoshop. A single auto contrast correction was applied to the entire blot, and no other manipulations were made to the image prior to scoring. Samples in lanes 1-7 correspond to the following total protein extracts: (1) Molecular Weight Marker (Western Sure; LI-COR #926-98000); (2) HEK293 Control; (3) HEK293 + 10 mM KCl (48 hours); (4) HEK293 + 50 mM KCl (48 hours); (5) HEK293 + 100 mM KCl (48 hours); (6) HEK293 + 100 mM NaCl (48 hours); (7) HEK293 + 100 mM NH_4Cl (48 hours); Note: The results from lane 7 were not discussed in the main manuscript, due to lack of complimentary data for NH_4Cl in related assays. However, NH_4Cl also induced NMD read-thru for CYP3A5*3 transcripts expressed in HEK293 cells but induced lower amounts of the 30 KDa $CYP3A5_{SV}$ protein compared to KCl or NaCl treatments.

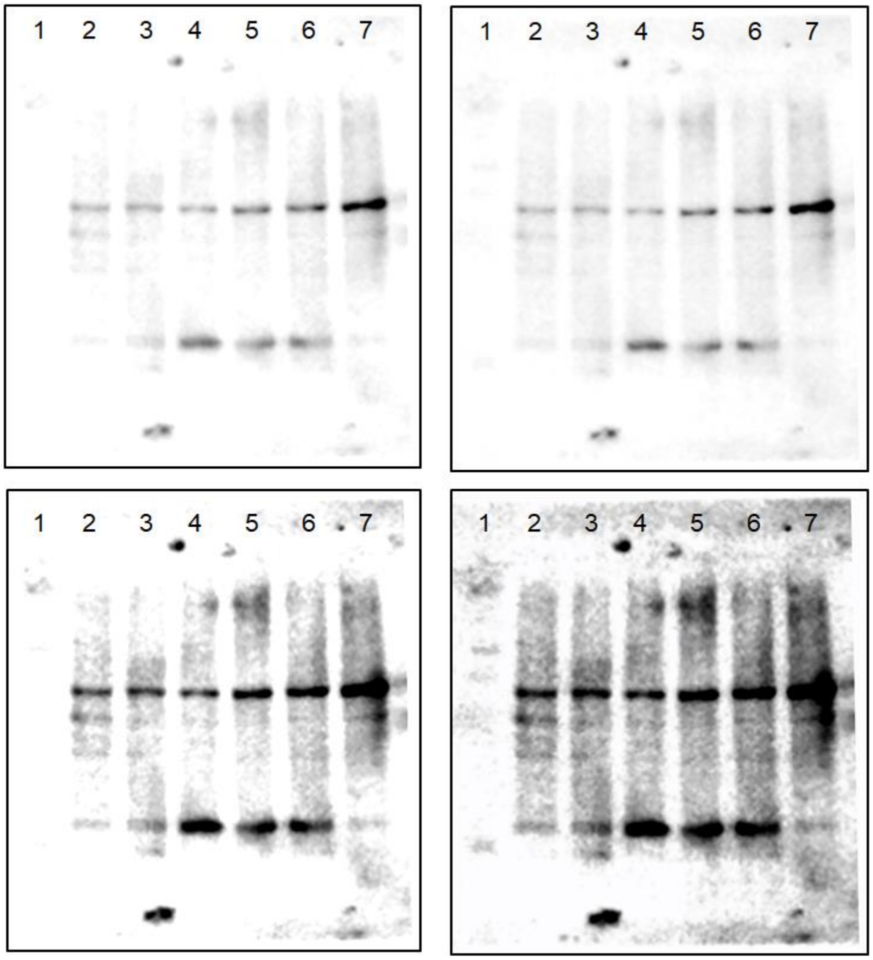

Supplemental Figure 6. Uncropped Western Blots for CYP3A5 at Multiple Exposures. Samples in lanes 1-7 in each image correspond to the following total protein extracts: (1) Molecular Weight Marker (Western Sure; LI-COR #926-98000); (2) HEK293 Control; (3) HEK293 + 10 mM KCl (48 hours); (4) HEK293 + 50 mM KCl (48 hours); (5) HEK293 + 100 mM KCl (48 hours); (6) HEK293 + 100 mM NaCl (48 hours); (7) HEK293 + 100 mM NH_4Cl (48 hours). Images were captured using the Azure Biosystems c600 imager and original unmodified scans captured using the autoscan feature were shown in Supplemental Figure 4. Images shown here represent increasing exposure the same gel taken between 15-240 seconds. Autoscan images were used for our analysis, after auto contrast adjustment was applied to the entire image in Adobe Photoshop.

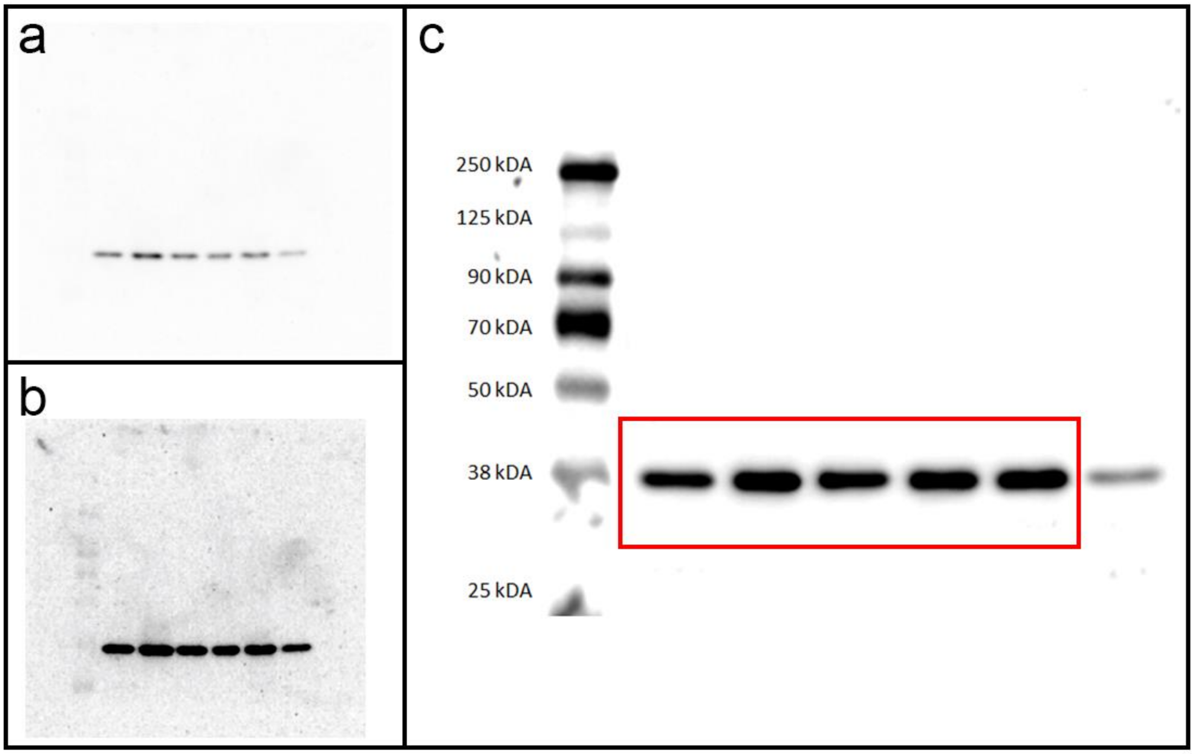

Supplemental Figure 7. Uncropped Western Blots for GAPDH at Multiple Exposures. Samples in lanes 1-7 in each image correspond to the following controls and total protein extracts: (1) Molecular Weight Marker (Western Sure; LI-COR #926-98000); (2) HEK293 Control; (3) HEK293 + 10 mM KCl (48 hours); (4) HEK293 + 50 mM KCl (48 hours); (5) HEK293 + 100 mM KCl (48 hours); (6) HEK293 + 100 mM NaCl (48 hours); (7) HEK293 + 100 mM NH_4Cl (48 hours). Images in panel **a.**) and **b.**) were captured using the Azure Biosystems c600 imager using 15 and 60 second exposures, respectively. **c**.) A composite image for the GAPDH blot was created to improve visibility of the molecular weight marker lane using an alternative exposure of the same blot, as described in Supplemental Figure 5. Cropped sections of the GAPDH blot shown in Figure 4a are highlighted in the red box.

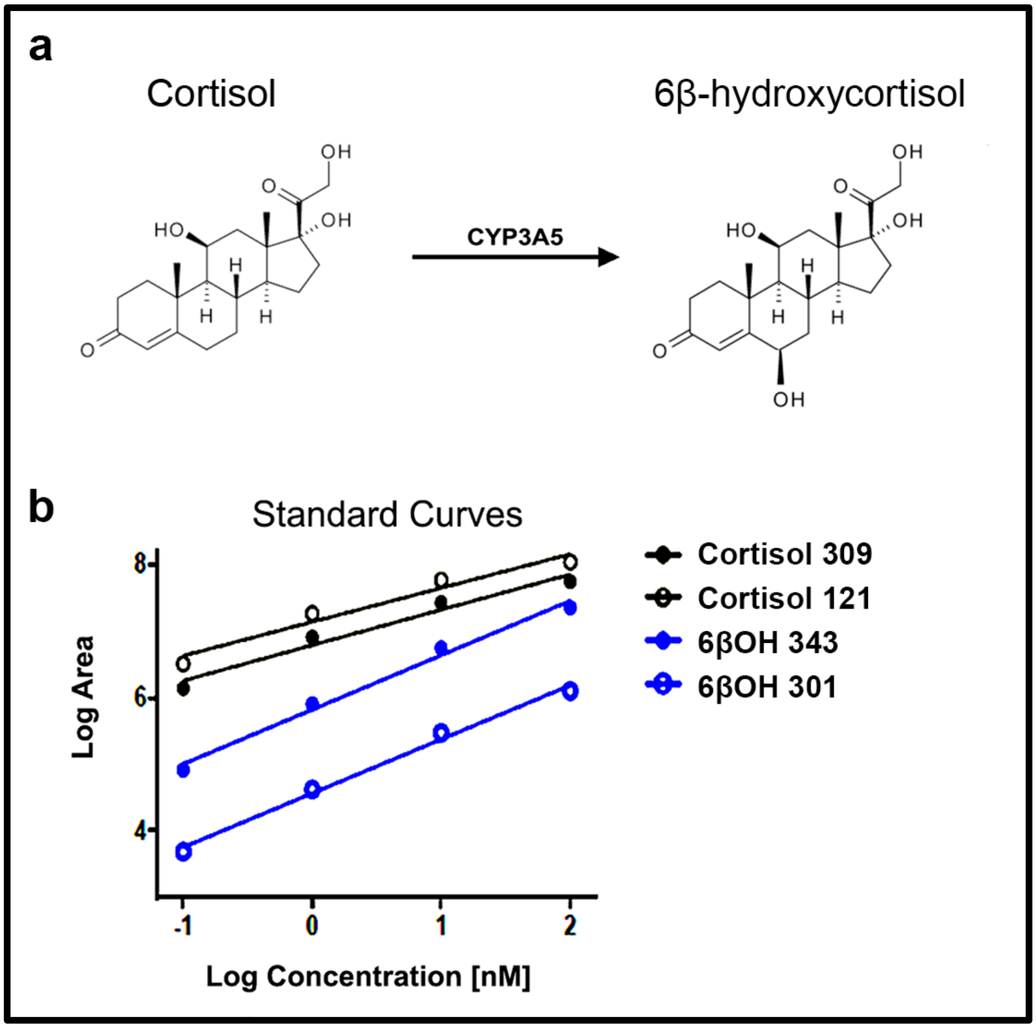

Supplemental Figure 8. LC/MS Standard Curves for Detecting the Conversion of Cortisol to 6β-hydroxycortisol in KCL-treated HEK293 cells. **a.**) Chemical structures of cortisol and the CYP3A5 metabolite, 6β-hydroxycortisol. **b.**) LC/MS standard curves for diagnostic fragment ions of cortisol and 6β-hydroxycortisol used in metabolic analysis. Drawings of cortisol adapted for this figure were obtained from https://commons.wikimedia.org/ and are in the public domain.

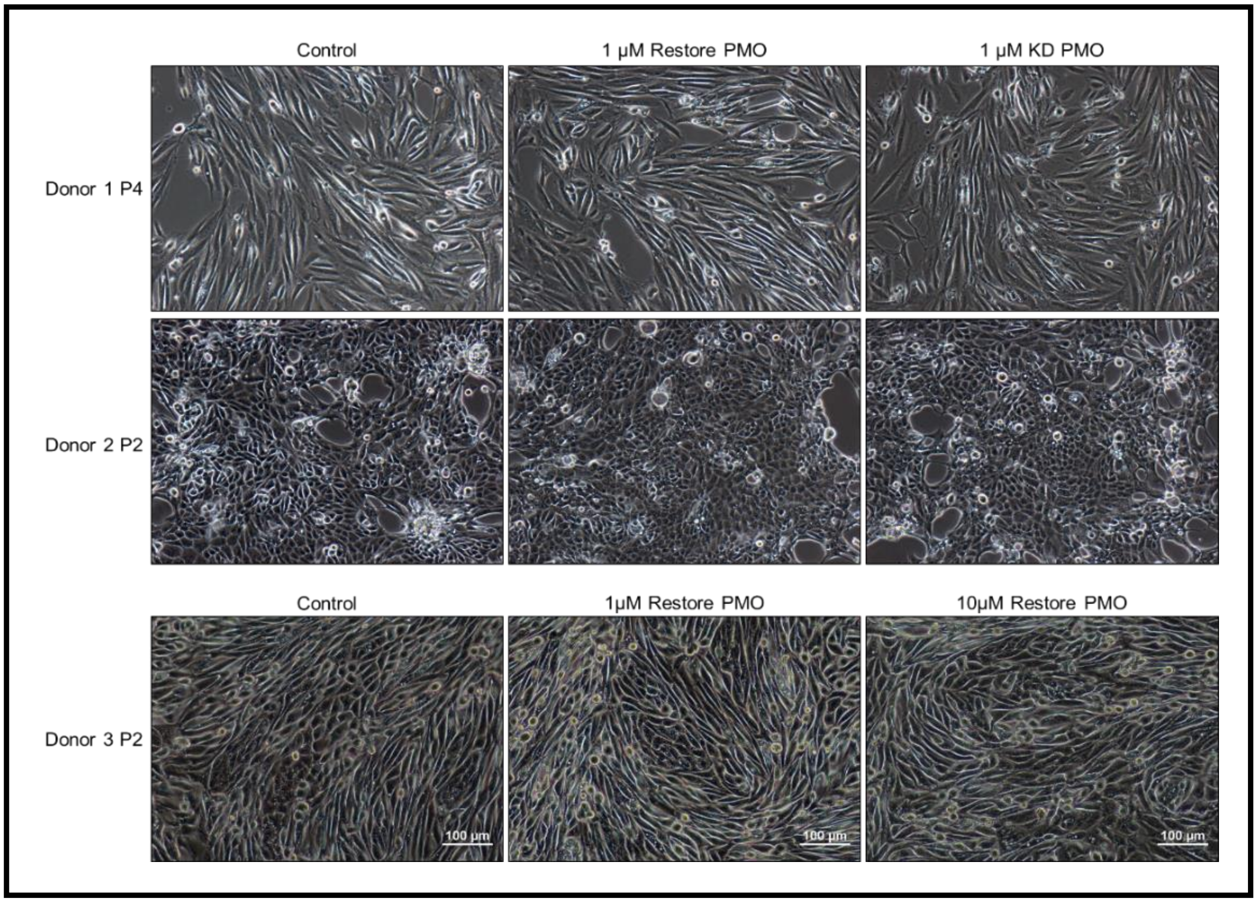

Supplemental Figure 9. PTEC morphology is unaffected by treatment with 3A5*3 or AUG PMOs. Phase contrast images of PTECs at the end of treatment with the restore (3A5*3) or knock down (KD) or AUG PMO shows no change in cell morphology across multiple PTEC donors. At higher passage, the cells take on an elongated morphology.

Supplemental Tables

Supplemental Table 1. Kidney Tissue Donor Demographics for PT-MPS Study.

Donor no.	Age	Sex	Ethnicity	Pre-existing conditions	Reason for nephrectomy
1	69	Male	White	N/A	Renal Cell Carcinoma
2	55	Male	White	Polyuria, Hyperkalemia, Polymyalgia Rheumatica	Renal Cell Carcinoma
3	62	Male	White	Gross hematuria, Hypertension, Obstructive Sleep Apnea, Depression	Clear Cell Renal Cell Carcinoma
4	63	Female	White	N/A	Unclassified Renal Cell Carcinoma
5	63	Male	White	Horseshoe Kidney	Oncocytoma
6	62	Male	White	Hypertension	Clear Cell Renal Cell Carcinoma
7	57	Male	White	N/A	Clear Cell Renal Cell Carcinoma
8	64	Male	White	Diverticulosis, Hyperlipidemia, Hypothyroid	Chromophobe Renal Cell Carcinoma
